# Wood-inhabiting fungal responses to forest naturalness vary among morpho-groups

**DOI:** 10.1038/s41598-021-93900-7

**Published:** 2021-07-16

**Authors:** Purhonen Jenna, Abrego Nerea, Komonen Atte, Huhtinen Seppo, Kotiranta Heikki, Læssøe Thomas, Halme Panu

**Affiliations:** 1 Biodiversity Unit, University of Turku, 20014 Turku, Finland; 2 Department of Biological and Environmental Science, University of Jyväskylä, P.O. Box 35, 40014 Jyväskylä, Finland; 3 School of Resource Wisdom, University of Jyväskylä, P.O. Box 35, 40014 Jyväskylä, Finland; 4 Department of Agricultural Sciences, University of Helsinki, P.O. Box 27, 00014 Helsinki, Finland; 5 Biodiversity Unit, Finnish Environment Institute, Latokartanonkaari 11, 00790 Helsinki, Finland; 6 Department of Biology/Natural History Museum of Denmark, University of Copenhagen, Universitetsparken 15, 2100 Copenhagen Ø, Denmark

**Keywords:** Ecology, Ecology

## Abstract

The general negative impact of forestry on wood-inhabiting fungal diversity is well recognized, yet the effect of forest naturalness is poorly disentangled among different fungal groups inhabiting dead wood of different tree species. We studied the relationship between forest naturalness, log characteristics and diversity of different fungal morpho-groups inhabiting large decaying logs of similar quality in spruce dominated boreal forests. We sampled all non-lichenized fruitbodies from birch, spruce, pine and aspen in 12 semi-natural forest sites of varying level of naturalness. The overall fungal community composition was mostly determined by host tree species. However, when assessing the relevance of the environmental variables separately for each tree species, the most important variable varied, naturalness being the most important explanatory variable for fungi inhabiting pine and aspen. More strikingly, the overall species richness increased as the forest naturalness increased, both at the site and log levels. At the site scale, the pattern was mostly driven by the discoid and pyrenoid morpho-groups inhabiting pine*,* whereas at the log scale, it was driven by pileate and resupinate morpho-groups inhabiting spruce. Although our study demonstrates that formerly managed protected forests serve as effective conservation areas for most wood-inhabiting fungal groups, it also shows that conservation planning and management should account for group- or host tree -specific responses.

## Introduction

Although the net loss rate of forest area has halved globally since the 1990’s, humans still alter forest ecosystems^1^. In the boreal region, forest management is the main reason for declines of dead-wood dependent species and concomitant changes in their community composition and structure^2–4^. Forest management, such as thinning or clear-cutting, decreases forest naturalness, and thus selects against species that require stable microclimate and continuous supply of dead wood^5,6^. At the resource level, management selects against species that require coarse dead wood or dead wood of economically unprofitable tree species (e.g. broadleaved trees in Finland)^2,3^.

Extant evidence about the fungal responses to forest management in the boreal zone comes from the limited number of tree species. Most studies have focused on Norway spruce (*Picea abies*, called henceforth spruce), even though pine (*Pinus* spp.) and broadleaved trees are also common^7–9^; particularly, broadleaved tree species have been neglected in boreal studies. This is problematic for forest management planning, because a large proportion of fungal species are specialized in certain host tree species^10^ and the consideration of different tree species in forest management often differs due to legislation or timber value. For example, in Finland the proportion of broadleaved trees in managed coniferous forests has been reduced by forestry practices, and in the case of European aspen (*Populus tremula*, called henceforth aspen), by browsing of large ungulates^11,12^. Fungal species inhabiting different tree species are also very likely to be adapted to different kind of environmental conditions^13^. Thus, it can be misleading to extrapolate management guidelines from studies on single tree species to all tree species.

Among the dead wood dependent organisms, fungi comprise a taxonomically and functionally diverse group regulating nutrient cycling^14–16^. Forest management reduces the diversity of wood-inhabiting fungi, especially affecting those species groups requiring large and well decayed logs which have drastically diminished due to forestry actions^17–19^. Decrease in the log size and in the amount of a particular dead wood type increase the probability of losing the species from the local community (e.g.^19–22^). Most studies assessing the effects of forest management on wood-inhabiting fungi have considered only those fungal groups that are relatively easy to detect and identify (mostly polypores and some corticioids and agarics). These groups comprise, however, a minor part of the total fungal species richness inhabiting deadwood (e.g.^23^). As wood-inhabiting fungal species differ in their ecological roles (e.g. which organic compounds they decompose^23^) as well as in their responses to changes in forest naturalness (e.g.^4,17,24^) there is a need to comprehensively assess how different fungal groups respond to forest naturalness.

Fungal traits, such as nutritional modes as well as fruitbody and spore characteristics, can affect species response to environmental changes and explain concomitant changes in species diversity^23,25–28^. Fungal fruitbodies function as a structure for spore (or other dispersal structure) formation and release in reproduction. Abrego et al.^26^ found that especially species with large, robust and long-lived fruitbodies suffer from forest management. They concluded that this was because of species with small fruitbodies were generally pioneers in the decay process, and larger ones were mid- and late successional specialists. Certain fruitbody morphologies have also been speculated to be adaptations to harsh environmental conditions, such as freezing, desiccation or excess moisture, which could lead to differences in group-specific responses^29,30^. Even though there is a vast variation in the morphology of wood inhabiting fungal fruitbodies^31^, compared to other groups such as animals and plants, very little is still known about how morphological traits modulate the responses of species to environmental variation^32,33^.

The overall aim of this study was to investigate how the level of forest naturalness, relates to the diversity of wood-inhabiting fungi on large logs in similar decay stages. For measuring forest naturalness, we calculated an index that jointly combined the variables of age of the canopy trees (increasing the index value), dead wood volume (increasing the index value) and number of stumps (decreasing the index value). We included all non-lichenized fungal groups producing fruitbodies. We surveyed large logs of the four dominant tree species in Fennoscandian boreal forests, namely birch (*Betula* spp.), spruce, Scots pine (*Pinus sylvestris*, hereafter pine) and aspen, in 12 seminatural forests with varying levels of naturalness. To disentangle the fungal groups-specific responses to environmental characteristics, we grouped the species according to their fruitbody morphology. Our specific aims were (1) to assess the relationship between forest naturalness as well as log characteristics and fungal species richness and composition, and (2) to evaluate whether the responses differ among morpho-groups and tree species. We hypothesized that (a) the species number and composition of groups that mostly occur on fine woody debris (as for example discoid and pyrenoid species) are less sensitive to a decrease in forest naturalness, as fine woody debris is in excess in the more managed sites^34^; (b) the species number and composition of groups with fruitbodies that are considered to better tolerate harsh environmental conditions (as for example pyrenoids) are less sensitive to a decrease in forest naturalness^29,30^; (c) the species number and composition of groups inhabiting pine and aspen are least sensitive to decreasing naturalness. The rationale of the latter hypothesis is that as pine and aspen are pioneer trees in boreal forests, the associated fungi can be expected to be more adapted to open canopy conditions^35^.

## Methods

### Study forests

**Figure 1:**
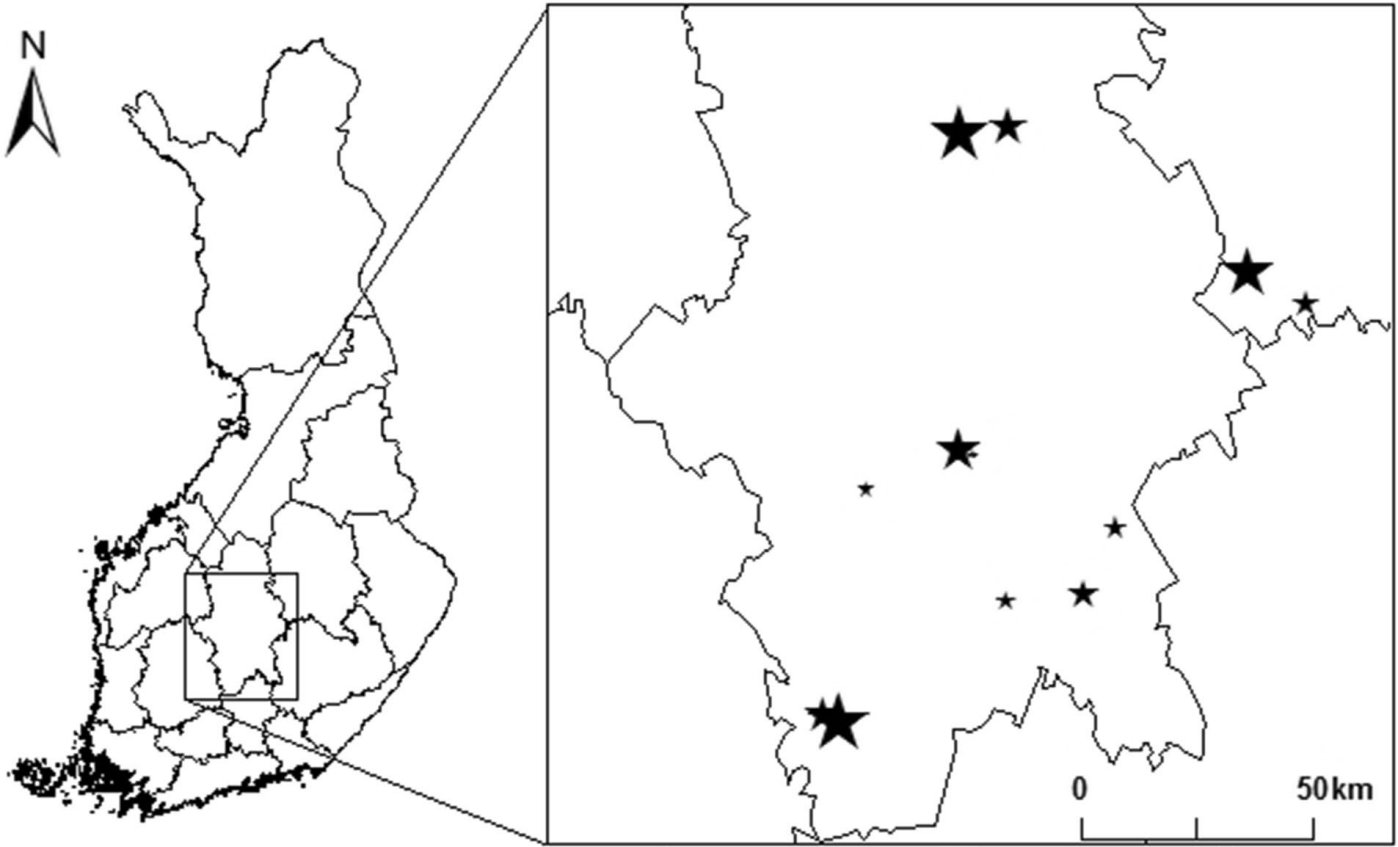
Location of the study sites in central Finland. The size of the symbol increases with forest naturalness. The lines represent the county borders. ArcMap, version 10.5.1^70^.

We selected 12 spruce-dominated forests with *Myrtillus* or *Oxalis-Myrtillus* forest site types^36^, located in the southern boreal zone^37^, central Finland (Fig. 1). We estimated the forest naturalness of each stand based on (1) the average age of the canopy trees in the focal 12 forest sites (data obtained from Metsähallitus, the State Forest Enterprise of Finland), (2) the average amount of dead wood (m^3^/ha), and 3) the average number of stumps per hectare. The latter two were estimated using randomly placed 50 m × 10 m transects. The number of transects varied from 4 to 8 per site: if the forest type among the log surroundings varied from the one considered typical for the site based on visual inspection, we established 2–4 additional transects to cover the within-site variation. From the transects all the dead wood units larger than 15 cm at the base diameter were measured for their length and the base and top diameter (to calculate their volume with the formula of a truncated cone), and the number of stumps was recorded. We were not able to separate the cut stumps from the natural stumps, as many of old stumps were heavily decayed. However, most of the natural stumps have adjacent fallen logs still visible, and in these cases, we did not consider them in the stump counting. Thus, majority of the counted stumps are cut stumps. Average values were calculated for each of the variables at the transect level. Those values were divided by 0.05 to estimate the average per hectare values. The average age of the canopy trees indicates climatic conditions (e.g. stability) of the forest^38^. The average dead wood amount indicates the substrate availability. The average number of cut stumps is related to intensity of the previous management. These variables greatly correlate with each other, for which reason we did not include them all simultaneously as separate variables into the analytical models. To overcome this problem, we derived an index, which summarized the variation of all the variables into a single variable. For calculating this index, we ranked each forest site according to the highest values in each of the variables, according to those values indicating highest naturalness (i.e. those that were oldest, had most dead wood volume per hectare and had least amount of stumps per hectare, see Table 1). For each of the variables, we gave as many points as their ranked order, and then summed these points to conform the naturalness index.

**Table 1:** The age of dominating forest cover in years, amount of deadwood (m^3^/ha), and number of stumps per hectare for each study site.

Site	Age/Deadwood/Stumps	Points	Index
Latokuusikko	173/334/0	11/12/12	35
Pyhä-Häkki	272/98/ 39	12/9/11	32
Kalajanvuori	140/100/64	9/10/10	29
Kuusimäki	140/171/110	8/11/6	25
Kivetty	132/86/103	6/8/8	22
Lortikka	150/32/96	10/1/9	20
Leivonmäki	135/67/135	7/6/4	17
Ilmakkamäki	124/65/117	5/5/5	15
Vuorilampi	116/81/199	3/7/3	13
Vaarunvuori	104/37/106	2/2/7	11
Hallinmäki	119/59/259	4/3/2	9
Tikkamäki	84/60/303	1/4/1	6

Corresponding forest naturalness index-value for each site is the sum of the points. The sites are sorted according to their Index-values from most to least natural.

### Study logs

We targeted our investigations to fungal communities in naturally fallen logs of the four most common tree species: birch, spruce, pine and aspen. In each forest site, four logs of each tree species were selected, totalling 16 logs in each forest, and 192 logs in the whole study (48 of each tree species). An exception to this was the site, Kettuvuori, where there were not enough suitable logs. To obtain the adequate number of study logs, we sampled additional logs from a nearby site, Tikkamäki. These forests are only 750 m apart from each other and separated by younger forests, thus they were treated as one site in the analyses. Given that the aim of the study was to study the effect of forest naturalness, we selected logs that were as uniform as possible in their qualities (in relation to their size, decay stage, bark cover and tree species) across the forest sites of varying naturalness. The criteria for selecting the logs are the following: (1) base diameter ≥ 15 cm, (2) decay stage 2–4^39^, and moss cover < 50%. We selected logs that were some distance from each other, but at few sites, this was not possible due to the small number of suitable logs. For each study log, we estimated the volume and decay stage in a similar way than described above for the site level dead wood measurements. The moss and bark cover were estimated as the proportion of the log surface area.

### Fungal data

Each study log was thoroughly examined for non-lichenized fruitbodies. All fruitbodies of a given taxa in a study log were regarded as one record of that taxa. Two surveys were conducted to account for temporal variation in fruitbody occurrence. The first survey was conducted in May–June and the second in August-October, which are the primary times for fruitbody formation^40–42^. Neither bark nor moss was removed during sampling. When possible, we identified fruitbodies to the species level in the field. However, most specimens were collected for microscopic identification. For some taxa, species-level identification was impossible, and thus the highest possible taxonomical or morpho-group status were assigned by unique annotations (e.g. *Mollisia* sp1, sp2 etc.). In some cases, these include entire species groups for which the taxonomy is poorly resolved. The nomenclature follows Index Fungorum^43^.

To study the relationship between forest naturalness and community structure of different morpho-groups, we separated the taxa into seven groups according to their fruitbody morphology; (1) gilled (fungi with fleshy pileus and stipe as well as pleurotoid fruitbodies without stipe, includes also *Gyromitra infula*), (2) discoid (hymenial layer in disc- or cup-like structure), (3) pileate (when mature, most of the fruitbody mass forms a hard pileus or is erected on the edges), (4) pyrenoid (hymenial layer inside peritechial structures, not embedded in stromatal layer), (5) branched (hymenial layer on branched structures), (6) resupinate (most of the fruitbody appressed to the substrate with exposed hymenium, can be slightly erected or pileate on the edges), (7) stromatoid (hymenial layer inside perithecia embedded in stromatic tissue).

### Analyses

#### Effects of forest naturalness on species richness

To model the site-level wood-inhabiting fungal species richness we fitted generalized linear models using Poisson regression. As the dependent variable, we considered the pooled number of fungal species per tree species within each site (called henceforth site-level species richness). As site-level explanatory variables, we included the naturalness index and as well as the tree-species specific mean volume of the study logs. The latter was included to account for the observation effort, as larger logs have more species. We did not include squared log volume, as the relationship of the mean log volume and species richness was linear.

To model the number of species per log (called henceforth log-level species richness) we fitted generalized linear mixed models using Poisson regression. In this case, we considered the log specific species richness as the dependent variable. The generalized linear mixed models included site-level as well as log-level explanatory variables. As the site-level explanatory variable, we included the naturalness index. The log-level explanatory variables included bark and moss coverage, decay stage, log volume and squared log volume. Prior to the analysis, the environmental variables were standardized by subtracting the mean and dividing it by the standard deviation. As the communities were situated on logs within the certain forest sites, site identity was considered a nested random effect. We estimated the model performance (pseudo-R-squared) with function “r.squaredGLMM” in the package “MuMIn”^44^.

All data analyses were conducted with R software version 3.4.3^45^. The generalized linear and mixed models were fitted using the “glmmTMB” function of the package “glmmTMB”^46^.

#### Effects of forest naturalness on community composition

We explored the relationship between log variables, forest naturalness, site identity and the fungal community composition using Nonmetric Multidimensional Scaling (NMDS). We used taxa’s presence-absence at the log level, and removed all taxa that occurred only once, as well as logs having a single fungal occurrence. The analysis was further conducted only for those morpho-groups that still included at least half of the surveyed logs after the above removal (96 for pooled data, and 24 for data split according to the tree species). Therefore, gilled and pileate groups on pine and aspen as well as branched and stromatoid groups on all tree species were excluded. To ensure that the NMDS stress level was under 20%, we chose the number of dimensions. Two- to four-dimensional scaling was performed depending on the group, with the function “metaMDS” of the package “vegan” using Bray–Curtis dissimilarities for each of the community pairs^47^.

To quantitatively assess the relationship between community composition and stand- and log-level characteristics, we additionally performed Spearman rank correlation analysis between community dissimilarities and different combinations of the Euclidean distances of the scaled explanatory variables using function “bioenv” of the “vegan” package^47^.

## Results

In total, we recorded 666 fungal taxa, of which 43% were resupinates, 23% discoids, 11% gilleds, 11% pyrenoids, 7.5% pileates, 3% stromatoids, and 1.5% branched (Supplementary Table S1). Altogether, there were 5764 fruitbody observations on the 192 studied logs. The data was dominated by rare taxa: 55% of the taxa occurred less than four times, 23% occurred 4–10 times, 20% occurred 11–50 times, and only 2% of the taxa occurred more than 50 times. All the most abundant taxa belonged to either discoid- or resupinate-group: *Mollisia* sp1. (169 records), *Orbilia delicatula* (122), *Botryobasidium subcoronatum* (105), *Peniophorella praetermissa* (95), *Hyaloscypha aureliella* (92) and the mycorrhizal *Amphinema byssoides* (92).

### Species richness along the forest naturalness gradient

**Figure 2:**
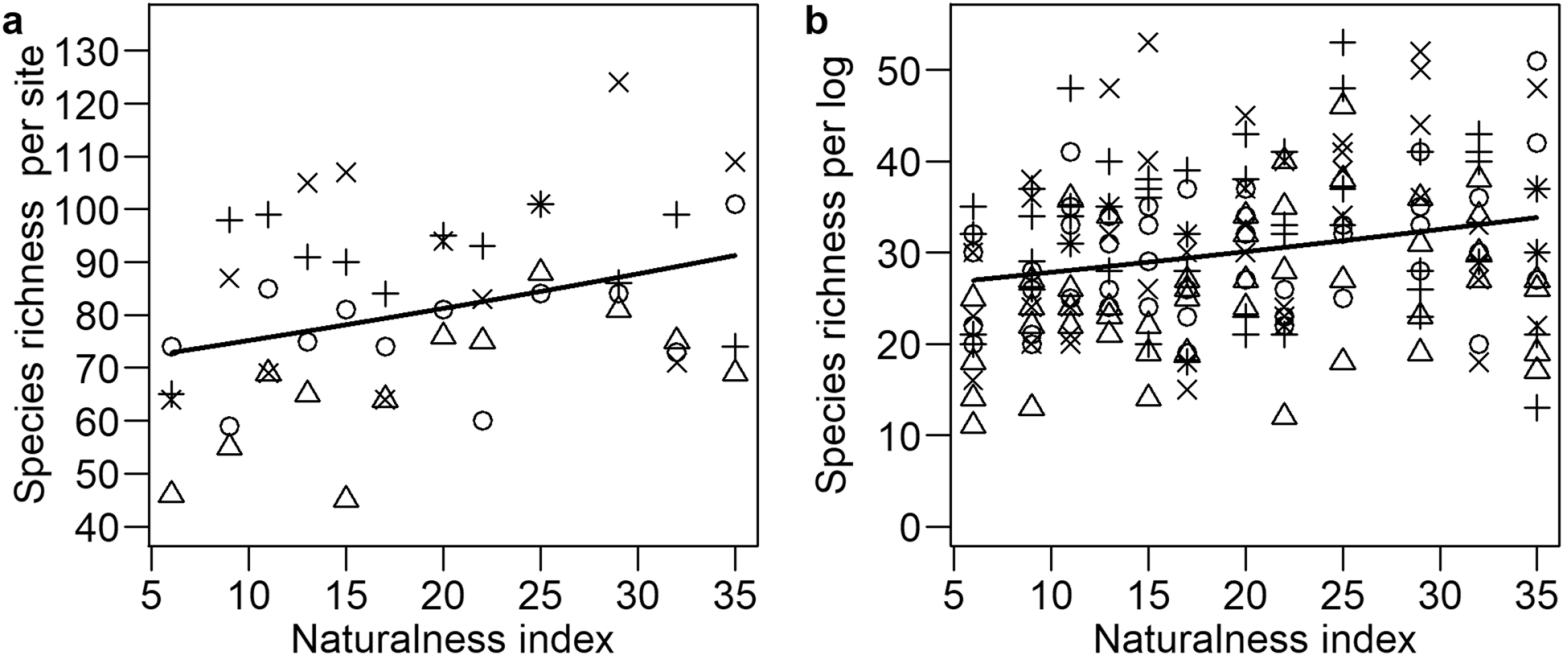
Species richness in relation to forest naturalness at the (**a**) site and (**b**) log levels. The lines are the regression between species richness and naturalness index. The different host tree species are indicated by different symbols, circles being spruce, triangles being pine, plusses being birch, and crosses being aspen.

The pooled number of taxa recorded from the six least natural forests was lower (S = 486 taxa) than that from the six most natural forest sites (S = 545). There was a positive relationship between the site-level species richness and forest naturalness (Fig. 2, Supplementary Table S2). This was mostly due to the strong responses of discoids and pyrenoids, especially those inhabiting pine. For most morpho-groups, there was no significant relationship with forest naturalness at the site level. The averaged study log volume had a positive relationship with species richness of most morpho-groups, being significant for the branched group inhabiting birch, gilled and resupinate groups inhabiting spruce, resupinates inhabiting pine, as well as gilled, resupinate and stromatoid groups inhabiting aspen.

The relationship of forest naturalness and log-level variables with the fungal species richness varied among morpho-groups and tree species (Table 2, Supplementary Table S3). Overall, naturalness increased species richness (Fig. 2), in which pileates and resupinates on spruce, pyrenoids on pine and branched taxa on aspen showed the strongest responses. The only variable that had a significant relationship with the species richness was decay stage, which negatively affected the diversity of pileates inhabiting birch. On spruce, moss coverage positively affected the diversity of gilled fungi and negatively the diversity of discoids. Bark coverage showed also a mixed pattern, with positive relationship with the pileate and branched groups inhabiting spruce and the pileates inhabiting aspen, but negative on resupinates inhabiting spruce and discoids inhabiting aspen. The increasing decay stage benefitted mostly resupinates. Log volume had a positive relationship with most of the groups, being significant for resupinates on pine and aspen and gilled on aspen. When studying the morpho-groups separately for different host tree species, most relationships were non-significant (Supplementary Table S3).

**Table 2:** Summarized estimate values from the generalized linear mixed models for log level species richness of all fungi and separately for different morpho-groups.

	All fungi		Gilled		Discoid		Pileate		Pyrenoid		Branched		Resupinate		Stromatoid	
Intercept	3.390	***	0.466	***	2.150	***	0.686	***	1.165	***	− 1.362	***	2.561	***	− 0.603	***
Bark	0.033	*	0.038		0.044	.	0.340	***	0.174	***	0.023		− 0.080	***	0.084	
Decay	− 0.024	.	− 0.010		− 0.094	***	− 0.074		− 0.005		0.062		0.039	.	− 0.367	**
Index	0.049	*	0.038		0.044	.	0.085		0.092	*	0.070		0.034		0.059	
Moss	0.016		0.223	***	− 0.029		0.037		− 0.136	**	− 0.128		0.054	*	0.038	
Volume	0.224	***	0.397	**	0.187	**	0.444	***	− 0.007		1.007	**	0.291	***	0.227	
Volume2	− 0.124	***	− 0.182		− 0.126	.	− 0.206	.	0.043		− 0.509	.	− 0.221	***	− 0.036	
R2m	0.353		0.188		0.136		0.269		0.164		0.090		0.323		0.086	
R2c	0.394		0.209		0.136		0.315		0.164		0.090		0.334		0.109	

The values correspond to the standardized explanatory variables of log bark cover (SD = 33.00), decay stage (0.54), naturalness index (9.00), moss cover (12.88), log volume (1.25) and log volume squared (1.66). Asterisk indicate P-values as follows: *** = *P* ≤ 0.000, ** = 0.000 < *P* ≤ 0.01, * = 0.01 < *P* ≤ 0.05, ∙ = 0.05 < *P* ≤ 0.1. The marginal (R2m) and conditional (R2c) coefficient of determination for Generalized mixed-effect models (Pseudo R-squared) are also reported.

### Species composition along the forest naturalness gradient

**Figure 3:**
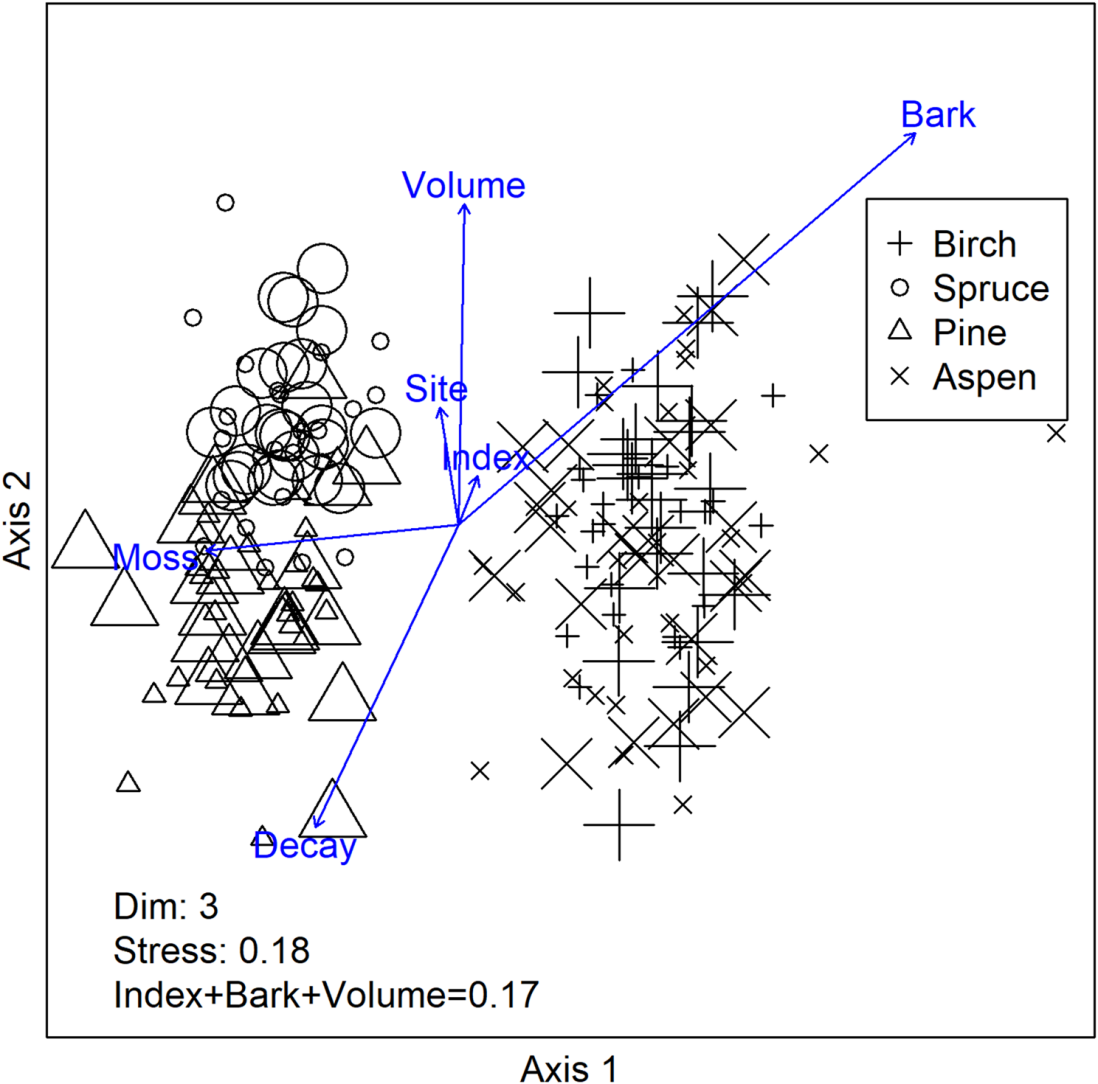
Non-metric multidimensional scaling of the fungal communities found in different tree species. The direction in which the environmental variables correlate with the fungal community similarity are indicated by the blue arrows, the length of which corresponds to the magnitude of the correlation. Small symbols correspond to the fungal communities from the six forest sites with lowest forest naturalness and the large symbols correspond to the fungal communities from the six forest sites with highest naturalness. The number of dimensions and stress for each scaling as well as the Spearman rank correlation coefficient for the combination of variables that received the highest correlation from the Bioenv-analysis are reported on the bottom of the figure. R software, version 3.4.3^45^.

The importance of forest naturalness in explaining community composition varied depending on the morpho-group and host tree species, being important mainly for fungal groups inhabiting pine and aspen (Supplementary Table S4, Supplementary Figure S1). Tree species was the most important driver of the overall fungal community composition (Fig. 3). The first axis clearly separated the fungal communities on broadleaved trees from conifers. The second axis indicated that communities on spruce and pine differed from each other, while the difference between birch and aspen was less clear. From the different combinations of explanatory variables included into the Bioenv-analysis, the combination of forest naturalness, log volume and bark cover showed the highest correlation (k = 0.17) with the overall community dissimilarities (Fig. 3).

## Discussion

Our study shows that forest naturalness (an index based on dead wood volume, number of cut stumps, and canopy tree age) positively correlated with the overall diversity of wood-inhabiting fungi. While similar results have previously been recorded for some wood-inhabiting groups (mostly polypores, agarics and corticioids)^6,17,19,20^, this is the first study providing a comprehensive assessment across major fruitbody-forming taxa inhabiting dead wood. Interestingly, the strength and direction of the relationship varied depending on the fungal group and/or host tree species.

At the log level, pileate and resupinate morpho-groups inhabiting spruce, pyrenoids on pine and branched fungi on aspen comprised the majority of the overall positive response in species richness to forest naturalness. A plausible explanation for this pattern could be that pileate fungi, especially those species specialized in advanced decay stages, are most dispersal and colonization limited and thus most sensitive to resource and microclimatic conditions varying with forest naturalness^4,35,48–51^. Our results indicate that also resupinate fungi inhabiting spruce and branched species on aspen follow the same patterns previously recorded for pileate fungi. We assumed the pyrenoid fungi would be less affected by decreasing forests naturalness, because they are able to reproduce on smaller dead wood items and have been hypothesized to be adapted to harsher environments^30^. However, our results do not support this hypothesis, and reveal the sensitivity of this poorly assessed group to forest naturalness.

Although we did not cover the full range of log characteristics (we selected the study logs to be as similar as possible between the study forests), we still found significant relationships between log characteristics and species richness. We found positive relationship between log volume and species richness of multiple morpho-groups. This finding is well demonstrated in previous studies, and is especially important for threatened species^21,34,52,53^. The fact that logs in more natural forest sites hold more species has highly relevant implications for forest conservation, as it highlights that conservation actions for wood-inhabiting fungi should not only focus on whether the required resources are present or not, but also on the larger scale characteristics of their habitat, that is the entire forest landscape.

We found a positive relationship between moss cover and the number of gilled fungi on spruce, while the relationship was negative with discoid group on spruce. Previously Heilmann-Clausen and Christensen^54^ found a positive relationship between the species richness of wood-inhabiting fungi and the moss cover of the logs. They suggested that moss cover indicates microclimatic conditions that enhance fungal growth. Increasing moss cover might also stabilise the microclimate of the decaying wood and thus advance fruitbody production^55^. We suggest, however, that the negative relationship with moss cover and discoid group could be because the fruitbodies are very small and could easily be covered by thick moss layer.

At the site level, the positive response of species richness to naturalness was mostly due to the fungal communities inhabiting pine, especially the morpho-groups with discoid and pyrenoid fruitbodies. This result may seem unexpected, as species inhabiting pine have generally been considered more tolerant to disturbances (e.g. forest fire), changes in microclimate and low substrate availability^35,56^. We argue that the observed pattern relates to the particular characteristics of aged pine trees in the least managed forests. Pine continues growing in diameter until the tree gets really old^57^, and thus very large pine trees are rare in managed forests. Although we aimed for logs of similar quality, we did not limit their maximum size; indeed, there was more variation in the sizes of pine study logs in most natural sites (Supplementary Results, Fig. S3). Furthermore, pine trees also gain special qualities and fungal communities when aging, especially when a pine grows and dies slowly and becomes a kelo tree, i.e. a dried barkless snag that remains standing for decades or centuries before falling to the ground^58,59^. Thus, the wood quality of a pine trees died young vs. old can be more different than that of birch, spruce and aspen, leading to a higher stand level diversity with increasing level of forest naturalness. This result underscores the importance of tree-specific management of fungal communities. Generally, forest management should target for as diverse tree species composition as possible. Pine is a special and more difficult case for management as it takes a millennium rather than centuries for a pine to reach total decomposition after its germination. One option would be to retain groups of pines at clear-cuts, and to assure their survival in the following cuttings.

The weak relationship between forest naturalness and the diversity of wood-inhabiting fungi likely results from the study design, which did not include the most intensively managed forests. In our study, the amount of large dead wood (> 15 cm in base diameter) at the least natural sites was as high as 60 m^3^/ha on average (Supplementary Results, Fig. S2), whereas typical managed boreal forests have less than 10 m^3^/ha^2,60^. Still, it was highly challenging to systematically find an equal number of study logs of different tree species, especially of broadleaved trees. Because for most morpho-groups there was neither significant positive nor negative relationship between species richness and naturalness at log or site levels, we conclude that semi-natural forests serve as efficient conservation areas for most wood-inhabiting fungi (see also^61^). It has been proposed that the minimum requirements for polypore conservation areas in boreal Europe are 20 hectare in size, having on the average 20 m^3^/ha of deadwood, mostly over 20 cm in DBH^62^. As we still found a weak positive relationship with naturalness, the previously reported thresholds do not seem to be able to fully protect fungal diversity and management guidelines should be revised accordingly.

The differences in the overall community composition were best explained by the host tree species, particularly the split between broadleaved and conifer trees, whereas naturalness explained only little. This result is in line with recent studies from the temperate Europe^16,63,64^, and it reinforces the idea that the tree species diversity should be taken into account in the conservation of wood-inhabiting fungal communities. The rather consistent importance of naturalness in explaining the species composition of fungal groups inhabiting pine and aspen indicates that fungal species inhabiting these tree species are more sensitive to changes caused by forest management, which is against our hypotheses. We assumed that the fungal groups inhabiting pine and aspen should be more resistant to changes in forest naturalness, because they likely were well- adapted to open canopy conditions^35^. For fungal groups inhabiting birch and spruce, different log characteristics were the most important determinants of community composition differences, and further the importance of different variables varied between the morpho-groups and the tree species. In previous studies, fungal community composition has been found to be shaped by epiphyte and bark cover^65^. For example, bark cover selects for species that are adapted to decay this specific resource^30,66^. Similarly, as with the positive relationship with species richness and moss cover, the increasing microclimatic stability or overgrowing effect with increasing moss cover may affect also the species composition.

Our results show that the morpho-groups with small fruitbodies require forest naturalness, as their diversity was positively influenced by the level of forest naturalness. This contradicts our hypothesis and findings of Abrego et al.^26^ who showed that the species with large, robust, long-lived fruitbodies suffer from forest management and species with small fruitbodies benefit from it. We did not classify the species according to the size of their fruitbodies; however, discoids and pyrenoids mainly have very small fruitbodies (typically few mm) compared to pileate polypores and corticioids (typically more than 5 cm). Another explanation of the different response to management might be that Abrego et al.^26^ studied also small diameter dead wood and compared highly contrasting forest sites in terms of forest naturalness, whereas our sites had smaller variability. In sum, fruitbody size may correlate with species susceptibility to forest naturalness but clearly, this does not apply to all fungal groups, tree species and geographical regions.

Finally, our study reveals that the taxonomic knowledge of many fungal groups is still poor. For example, we found many species that are new to Finland, some that are new to Europe and several that are new to science^67,68^. Molecular methods would have likely increased the number of taxa discovered in our study through verification of species ID´s or observing species that were not fruiting at the time of the samplings^69^. In addition, this would have probably increased the separation power of our analyses as some patterns in the community composition might have been hampered by our inability to separate some grouped taxa.

## Conclusions

Currently, conservation guidelines of wood-inhabiting fungi are mainly based on polypores^50^. Because other wood-inhabiting fungal groups may respond differently to forest naturalness, current guidelines may not be adequate in protecting fungal diversity. The varying responses of different morpho-groups on different host trees imply that it is important to consider also the understudied fungal groups, such as groups with discoid and pyrenoid fruitbodies, and tree species in management and conservation planning. Importantly, our study pointed out that the dead wood of old and large pines, only found in the most natural boreal forests, is crucial for the most poorly known fungal groups, such as fungi with discoid fruitbodies.

## Supplementary Information

Supplementary Figure S1.

Supplementary Table S1.

Supplementary Table S2.

Supplementary Table S3.

Supplementary Table S4.

Supplementary Information.

Supplementary Legends.
