# Supplementary material for: Wood-inhabiting fungal responses to forest naturalness vary among morpho-groups
Source: Sci Rep. 2021 Jul 16;11:14585. doi: 10.1038/s41598-021-93900-7 (PMC8285386; doi:10.1038/s41598-021-93900-7)
Supplement: Supplementary file 1 — Supplementary Figure S1. [file 41598_2021_93900_MOESM1_ESM.pdf]

Wood-inhabiting fungal responses to forest naturalness vary among morpho-groups

Supplementary Figure S1

Purhonen Jenna, Abrego Nerea, Komonen Atte, Huhtinen Seppo, Kotiranta Heikki, Læssøe Thomas & Halme Panu

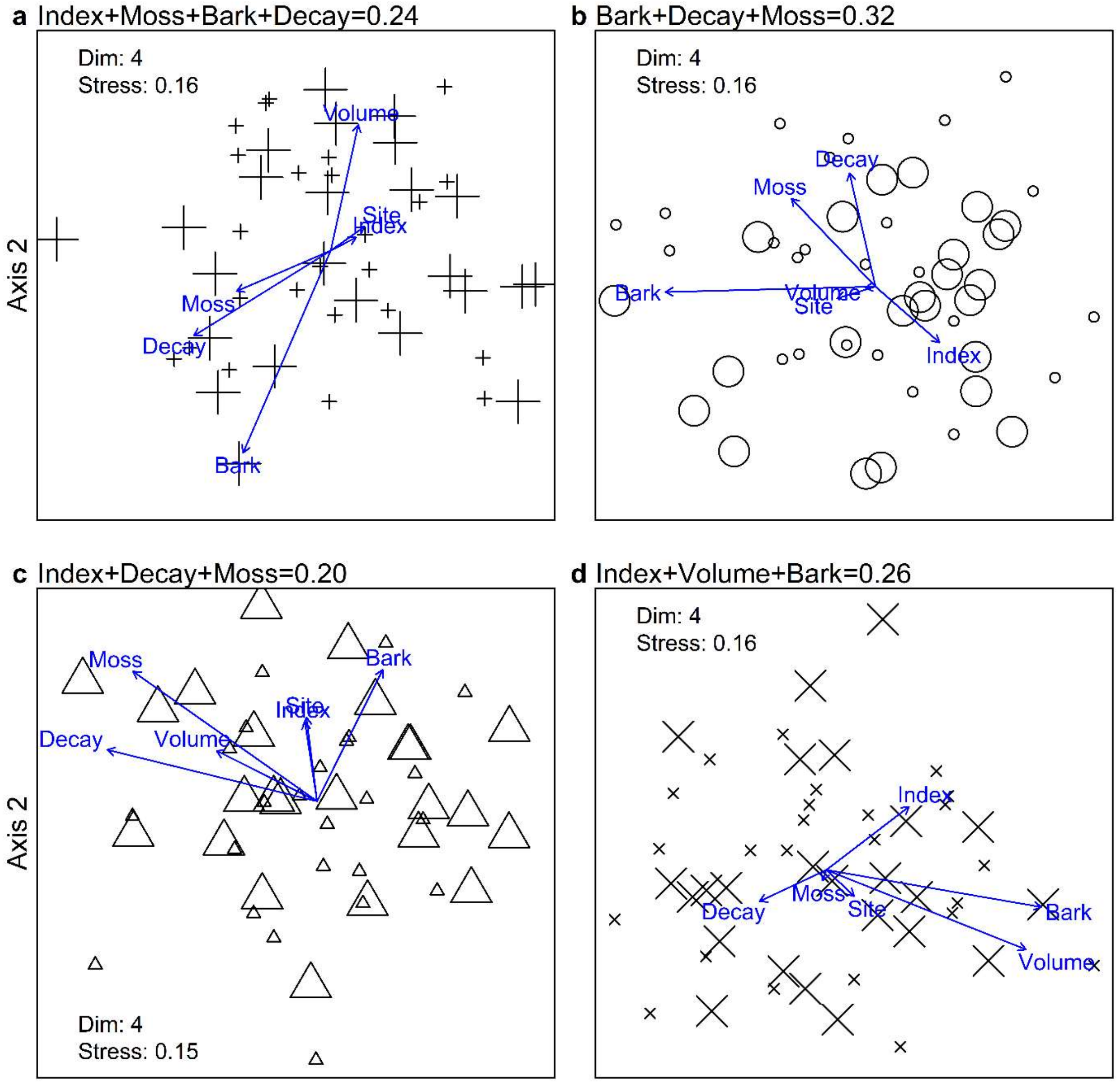

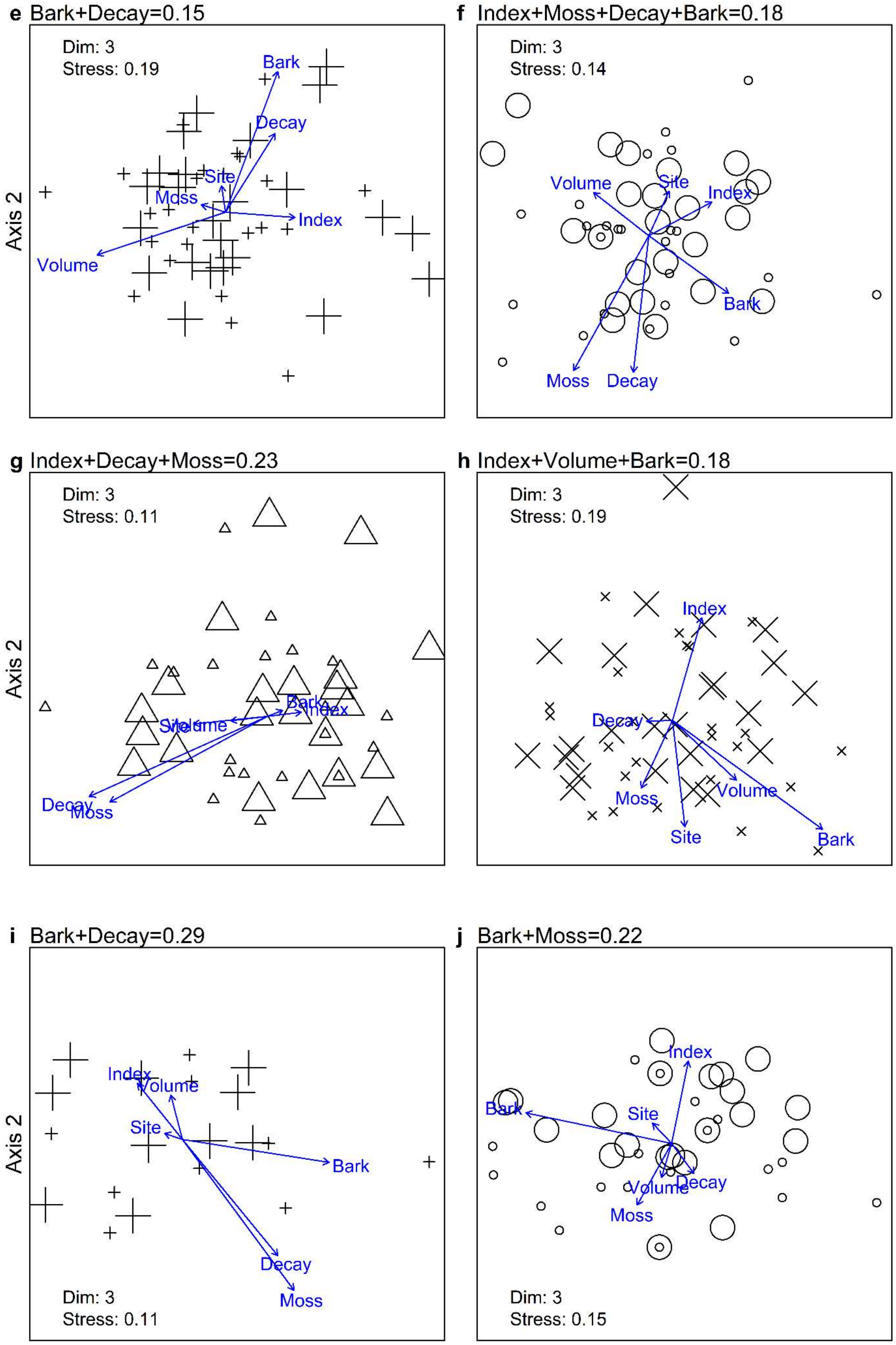

e Bark+Decay=0.15
Dim: 3
Stress: 0.19
Bark
Decay
Site
Moss
Index
Volume
Axis 2
f Index+Moss+Decay+Bark=0.18
Dim: 3
Stress: 0.14
Volume
Site
Index
Bark
Moss
Decay
g Index+Decay+Moss=0.23
Dim: 3
Stress: 0.11
Bark
Index
Site
Volume
Decay
Moss
Axis 2
h Index+Volume+Bark=0.18
Dim: 3
Stress: 0.19
Index
Decay
Moss
Volume
Site
Bark
i Bark+Decay=0.29
Index
Volume
Site
Bark
Decay
Moss
Dim: 3
Stress: 0.11
Axis 2
j Bark+Moss=0.22
Index
Site
Bark
Volume
Decay
Moss
Dim: 3
Stress: 0.15

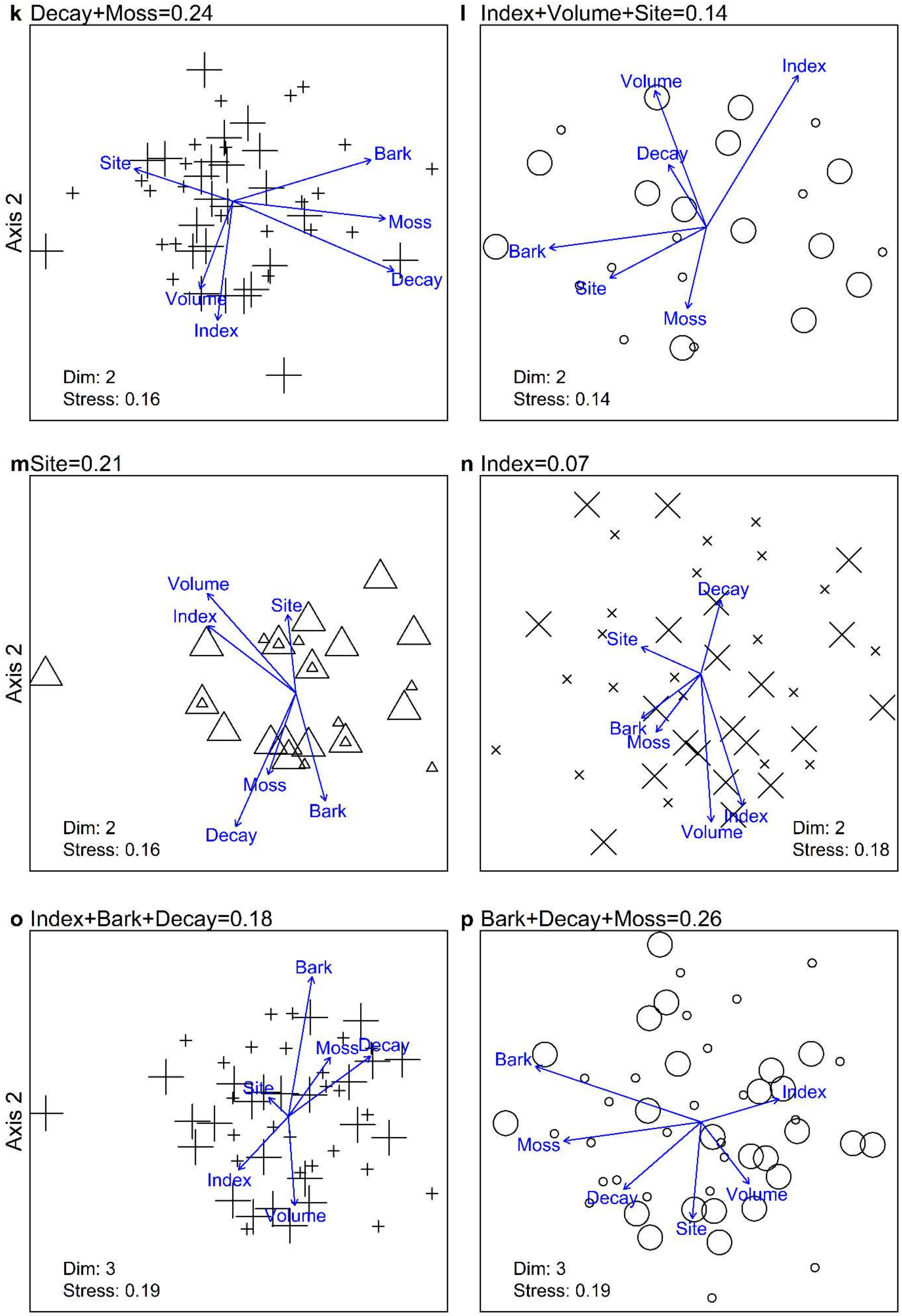
k Decay+Moss=0.24
Site
Bark
Moss
Decay
Volume
Index
Dim: 2
Stress: 0.16
Axis 2
l Index+Volume+Site=0.14
Volume
Index
Decay
Bark
Site
Moss
Dim: 2
Stress: 0.14
m Site=0.21
Volume
Index
Site
Moss
Bark
Decay
Dim: 2
Stress: 0.16
Axis 2
n Index=0.07
Decay
Site
Bark
Moss
Index
Volume
Dim: 2
Stress: 0.18
o Index+Bark+Decay=0.18
Bark
Moss
Decay
Site
Index
Volume
Dim: 3
Stress: 0.19
Axis 2
p Bark+Decay+Moss=0.26
Bark
Index
Moss
Decay
Volume
Site
Dim: 3
Stress: 0.19

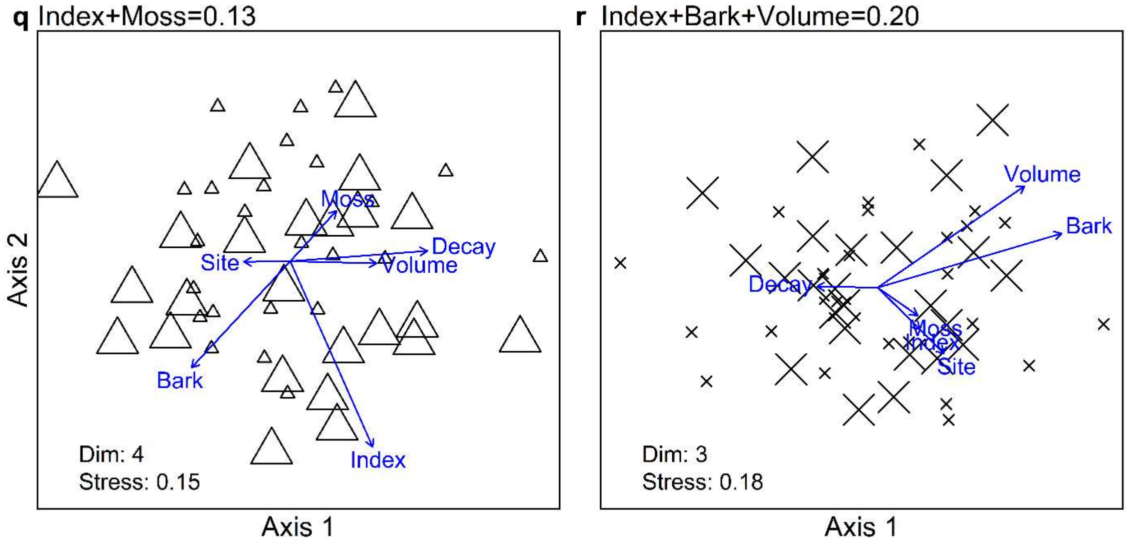

Figure S1. Non-metric multidimensional scaling (NMDS) for the total fungal communities on a) birch, b) spruce, c) pine, and d) aspen. Panels e-r show the NMDS applied to different morpho-groups and different tree species: (1) discoids on e) birch, f) spruce, g) pine and, h) aspen, (2) pileates on i) birch and, j) spruce, (3) pyrenoids on k) birch, l) spruce, m) pine and n) aspen, and (4) resupinates on o) birch, p) spruce, q) pine and r) aspen. The increasing gradient for the environmental variables are indicated by the blue arrows, the length of which corresponds to the magnitude of the correlation between the variable and the scaling. The text above the figure reports the Spearman rank correlation coefficient for the combination of variables that received the highest correlation from the Bioenv-analysis. Small symbols indicate the communities from the six forest sites with least naturalness and the large symbols indicate the communities from the six forest sites with highest naturalness. The number of dimensions and stress for each scaling is also reported in the figure legend.
