# Supplementary material for: Wood-inhabiting fungal responses to forest naturalness vary among morpho-groups
Source: Sci Rep. 2021 Jul 16;11:14585. doi: 10.1038/s41598-021-93900-7 (PMC8285386; doi:10.1038/s41598-021-93900-7)
Supplement: Supplementary file 2 — Supplementary Table S1. [file 41598_2021_93900_MOESM2_ESM.pdf]

Wood-inhabiting fungal responses to forest naturalness vary among morpho-groups

Supplementary Table S1

Purhonen Jenna, Abrego Nerea, Komonen Atte, Huhtinen Seppo, Kotiranta Heikki, Læssøe Thomas, & Halme Panu

Table S1. List of species or taxonomic groups detected in the study in alphabetical order. Morpho-group refers to the classification of the detected taxa based on their fruit body morphology. Abundance refers to the total number of logs that the taxa were observed (maximum number of logs in the study was 192).

Taxa	Morpho-group	Abundance
Acanthostigma sp1.	Pyrenoid	2
Acrogenospora carmichaeliana	Pyrenoid	1
Actidium hysterioides	Pyrenoid	13
Alutaceodontia alutacea	Resupinate	13
Amphinema byssoides	Resupinate	92
Amphisphaerella dispersella	Pyrenoid	1
Amphisphaeria bertiana	Pyrenoid	1
Amylocorticiellum cremeoisabellinum	Resupinate	1
Amylocorticiellum subillaqueatum	Resupinate	1
Amylocorticium cebennence	Resupinate	2
Amylocorticium pedunculatum	Resupinate	1
Amylocystis lapponica	Pileate	3
Amyloporia sinuosa	Resupinate	22
Amylostereum chailletii	Pileate	8
Amyloxenasma grisellum	Resupinate	5
Annulohypoxylon multiforme	Stromatoid	20
Antrodia albobrunnea	Resupinate	1
Antrodia macra	Resupinate	3
Antrodia pulvinascens	Resupinate	1
Antrodia serialis	Pileate	40
Antrodia xantha	Resupinate	10
Antrodiella pallescens	Resupinate	1
Antrodiella romellii	Resupinate	2
Aphanobasidium pseudotsugae	Resupinate	36
Arachnopeziza delicatula	Discoid	28
Arachnopeziza cf aranea	Discoid	2
Arachnopeziza leonina	Discoid	33
Arachnopeziza sp nov.	Discoid	1
Arachnopeziza sp1.	Discoid	2
Arachnopeziza sp2.	Discoid	1
Arachnopeziza sp3.	Discoid	1
Armillaria borealis	Gilled	2
Arrhenia epichysium	Gilled	1
Artomyces cristatus	Branched	1
Artomyces pyxidatus	Branched	6
Ascocorticium anomalum	Resupinate	2
Ascocoryne cylichnium	Discoid	75
Ascocoryne sarcoides	Discoid	17

Asterodon ferruginosus	Resupinate	5
Asterostroma laxum	Resupinate	1
Athelia acrospora	Resupinate	2
Athelia decipiens	Resupinate	51
Athelia epiphylla coll	Resupinate	6
Athelia neuhoffii	Resupinate	25
Athelopsis glaucina	Resupinate	1
Athelopsis subinconspicua	Resupinate	14
Auricularia auricula-judae	Discoid	2
Basidiodendron caesiocinereum	Resupinate	9
Basidiodendron cinereum	Resupinate	4
Basidioradulum crustosum	Resupinate	9
Bertia moriformis	Pyrenoid	53
Bisporella citrina	Discoid	49
Bjerkandera adusta	Pileate	3
Boidinia furfuracea	Resupinate	2
Bolbitius reticulatus	Gilled	1
Boliniaceae sp1.	Pyrenoid	3
Botryobasidium botryosum	Resupinate	78
Botryobasidium conspersum	Resupinate	3
Botryobasidium intertextum	Resupinate	7
Botryobasidium laeve	Resupinate	3
Botryobasidium medium	Resupinate	5
Botryobasidium obtusisporum	Resupinate	1
Botryobasidium subcoronatum	Resupinate	105
Botryohypochnus isabellinus	Resupinate	31
Butyrea luteoalbum	Resupinate	13
Byssomerulius corium	Pileate	1
Byssoporia terrestris	Resupinate	3
Cabalodontia bresadolae	Resupinate	1
Cabalodontia cretacea	Resupinate	17
Cabalodontia subcretacea	Resupinate	2
Calocera cornea	Branched	11
Calocera furcata	Branched	12
Calocera viscosa	Branched	1
Calycellina guttulifera	Discoid	2
Calycellina ochracea	Discoid	5
Calycellina sp1.	Discoid	1
Calyptella sp1.	Discoid	4
Camarops lutea/pugillus complex	Stromatoid	2
Camarops tubulina	Stromatoid	2
Capitotricha bicolor	Discoid	12
Capronia cf mansonii	Pyrenoid	1
Capronia cf pilosella	Pyrenoid	20
Capronia cf semi-immersa	Pyrenoid	1
Capronia sp4.	Pyrenoid	10
Capronia sp5.	Pyrenoid	4
Ceraceomyces eludens	Resupinate	24

Ceraceomyces microsporus	Resupinate	18
Ceraceomyces serpens	Resupinate	9
Ceraceomyces tessulatus	Resupinate	11
Ceratosebacina longispora	Resupinate	1
Ceratosphaeria cf subferruginea	Pyrenoid	1
Ceratosphaeria lampadophora	Pyrenoid	3
Ceratosphaeria rhenana	Pyrenoid	30
Ceratostomella rostrata	Pyrenoid	5
Cerinomyces crustulinus	Resupinate	10
Cerioporus leptocephalus	Pileate	2
Cerioporus mollis	Pileate	7
Ceriporia excelsa	Resupinate	3
Ceriporia reticulata	Resupinate	1
Ceriporia viridans	Resupinate	3
Ceriporiopsis resinascens	Resupinate	5
Cerrena unicolor	Pileate	1
Chaetoderma luna	Resupinate	6
Chaetosphaeria cf cupulifera	Pyrenoid	11
Chaetosphaeria myriocarpa	Pyrenoid	1
Chaetosphaeria sp1.	Pyrenoid	10
Chaetosphaeria sp2.	Pyrenoid	1
Chaetosphaeria vermicularioides	Pyrenoid	2
Cheimonophyllum candidissimum	Gilled	15
Chlorencoelia versiformis	Discoid	3
Chlorociboria aeruginascens	Discoid	17
Chlorociboria aeruginosa	Discoid	1
Chrysomphalina chrysophylla	Gilled	1
Ciliolarina aff pinicola	Discoid	2
Ciliolarina cf laetifica	Discoid	6
Ciliolarina concortica	Discoid	2
Ciliolarina neglecta	Discoid	21
Ciliolarina sp1.	Discoid	1
Cinereomyces lindbladii	Resupinate	1
Cistella cf geelmyedenii	Discoid	1
Cistella cf improvisa	Discoid	5
Cistella cf microspora	Discoid	1
Cistella sp1.	Discoid	2
Cistella sp2.	Discoid	1
Cistella sp3.	Discoid	1
Cistella sp4.	Discoid	1
Cistella sp5.	Discoid	1
Cistella sp6.	Discoid	1
Cistella sp8.	Discoid	1
Claussenomyces atrovirens	Discoid	31
Clavulicium delectabile	Resupinate	1
Colacogloea peniophorae	Resupinate	1
Conferticium ochraceum	Resupinate	3
Conferticium ravum	Resupinate	1

Coniochaeta subcorticalis	Pyrenoid	1
Coniophora arida	Resupinate	13
Coniophora olivacea	Resupinate	43
Coniophora puteana	Resupinate	10
Coronicium alboglaucum	Resupinate	1
Coronophora sp nov	Pyrenoid	2
Corticium boreoroseum	Resupinate	1
Corticium polygonioides	Resupinate	6
Corticium roseum	Resupinate	7
Crepidotus calolepis	Gilled	5
Crepidotus cesatii	Gilled	5
Crepidotus pallidus	Discoid	16
Crepidotus subverrucisporus	Gilled	1
Crocicreas sp1.	Discoid	1
Crustoderma corneum	Resupinate	1
Crustoderma dryinum	Resupinate	1
Crustoderma efibulatum	Resupinate	1
Cryptodiscus foveolaris	Discoid	1
Cryptodiscus muuri-itiö	Discoid	5
Cryptodiscus pallidus	Discoid	1
Cryptodiscus pini	Discoid	10
Cudonia confusa	Gilled	1
Cyathicula sp1.	Discoid	2
Cyathicula sp2.	Discoid	1
Cylindrobasidium evolvens	Resupinate	8
Cystoderma jasonis	Gilled	2
Dacrymyces adpressus	Discoid	1
Dacrymyces lacrymalis	Discoid	8
Dacrymyces macnabbii	Discoid	16
Dacrymyces microsporus	Discoid	16
Dacrymyces minor	Discoid	16
Dacrymyces minutus	Discoid	9
Dacrymyces ovisporus	Discoid	2
Dacrymyces sp1.	Discoid	1
Dacrymyces sp2.	Discoid	1
Dacrymyces stillatus	Discoid	34
Dacrymyces tortus	Discoid	24
Dacryobolus karstenii	Resupinate	5
Dacryobolus sudans	Resupinate	5
Daldinia concentrica	Stromatoid	1
Dialonectria cf episphaeria	Pyrenoid	6
Diatrype stigma	Stromatoid	1
Diatrypella sp1.	Stromatoid	1
Dichostereum boreale	Resupinate	1
Discomycetes sp2.	Discoid	1
Discomycetes sp3.	Discoid	2
Discomycetes sp5.	Discoid	1
Ditiola peziziformis	Discoid	1

Durella melanochlora	Discoid	13
Echinosphaeria canescens	Pyrenoid	3
Echinosphaeria cincinnata	Pyrenoid	4
Elmerina caryae	Resupinate	4
Endoxyla macrostoma	Pyrenoid	1
Endoxyla parallela	Stromatoid	11
Endoxyla rostrata	Pyrenoid	4
Entoloma depluens	Gilled	3
Eutypa flavovirens	Stromatoid	7
Exidia glandulosa	Discoid	6
Exidia repansa	Discoid	3
Exidia saccharina	Discoid	1
Exidiopsis calcea	Resupinate	1
Exidiopsis effusa	Resupinate	1
Flagelloscypha sp1.	Discoid	1
Flammulaster limulatus	Gilled	12
Flaviporus citrinellus	Resupinate	3
Fomes fomentarius	Pileate	45
Fomitopsis betulina	Pileate	2
Fomitopsis pinicola	Pileate	73
Fomitopsis rosea	Pileate	3
Galerina hypnorum	Gilled	2
Galerina marginata	Gilled	7
Galerina mniophila	Gilled	3
Galerina pumila	Gilled	1
Galerina stylifera	Gilled	5
Galzinia incrustans coll	Resupinate	10
Ganoderma applanatum	Pileate	1
Gelatoporia dichrous	Pileate	1
Globulicium hiemale	Resupinate	37
Gloeocystidiellum convolvens	Resupinate	6
Gloeocystidiellum leucoxanthum	Resupinate	5
Gloeocystidiellum luridum	Resupinate	2
Gloeocystidiellum porosum	Resupinate	3
Gloeodontia subasperispora	Resupinate	2
Gloeophyllum sepiarium	Pileate	1
Gloeoporus pannocinctus	Resupinate	8
Gloeoporus taxicola	Resupinate	3
Gloiothele citrina	Resupinate	14
Glonium nitidum	Pyrenoid	2
Godronia urceolus	Discoid	1
Gorgoniceps aridula	Discoid	1
Gorgoniceps hypothallosa	Discoid	6
Gymnopilus penetrans	Gilled	35
Gymnopilus picreus	Gilled	7
Gymnopus androsaceus	Gilled	6
Gymnopus confluens	Gilled	1
Gymnopus dryophilus	Gilled	2

Gyromitra infula	Gilled	5
Haavankaarnapyr	Pyrenoid	2
Hamatocanthoscypha laricionis	Discoid	1
Hamatocanthoscypha sp nov	Discoid	1
Hamatocanthoscypha sp1.	Discoid	1
Hamatocanthoscypha sp2.	Discoid	5
Hamatocanthoscypha sp3.	Discoid	1
Hamatocanthoscypha straminella	Discoid	6
Helicobasidium sp1.	Resupinate	1
Helminthosphaeria aff carpathica	Pyrenoid	2
Helminthosphaeria aff odontiae	Pyrenoid	2
Helminthosphaeria aff pilifera	Pyrenoid	1
Helminthosphaeria cf gibberosa	Pyrenoid	4
Helminthosphaeria ludens	Pyrenoid	8
Helminthosphaeria sp1.	Pyrenoid	1
Helminthosphaeriaceae sp nov.	Pyrenoid	8
Helvella macropus	Gilled	1
Hemimycena sp1.	Gilled	1
Henningsomyces candidus	Discoid	15
Henningsomyces pienikarva	Discoid	2
Hericium cirrhatum	Pileate	1
Hericium coralloides	Branched	1
Hilberina aff moseri	Pyrenoid	1
Hilberina aff munkii	Pyrenoid	2
Hilberina cf caudata	Pyrenoid	3
Humaria hemisphaerica	Discoid	11
Hyalopeziza millepunctata	Discoid	5
Hyaloscypha albohyalina	Discoid	12
Hyaloscypha aureliella	Discoid	92
Hyaloscypha diabolica	Discoid	1
Hyaloscypha epiporia	Discoid	3
Hyaloscypha fuckelii	Discoid	38
Hyaloscypha intacta	Discoid	24
Hyaloscypha latispora	Discoid	1
Hyaloscypha leuconica	Discoid	22
Hyaloscypha quercicola	Discoid	1
Hyaloscypha sp1. nov.	Discoid	1
Hyaloscypha spiralis	Discoid	10
Hyaloscypha vitreola	Discoid	28
Hymenochaete fuliginosa	Resupinate	3
Hymenochaetopsis tabacina	Pileate	2
Hymenoscyphus sp2.	Discoid	1
Hymenoscyphus sp3.	Discoid	1
Hymenoscyphus vikgultorum	Discoid	1
Hyphoderma cremeoalbum	Resupinate	2
Hyphoderma definitum	Resupinate	9
Hyphoderma incrustatum	Resupinate	6
Hyphoderma obtusiforme	Resupinate	1

Hyphoderma occidentale	Resupinate	4
Hyphoderma roseocremeum	Resupinate	1
Hyphoderma setigerum	Resupinate	29
Hyphoderma sibiricum	Resupinate	1
Hyphodiscus hemiamyloideus	Discoid	18
Hyphodiscus hymeniophilus	Discoid	2
Hyphodontia abieticola	Resupinate	9
Hyphodontia alutaria	Resupinate	2
Hyphodontia barba-jovis	Resupinate	5
Hyphodontia curvispora	Resupinate	1
Hyphodontia efibulata	Resupinate	2
Hyphodontia pallidula	Resupinate	18
Hyphodontia subalutacea	Resupinate	35
Hypholoma fasciculare	Gilled	1
Hypholoma polytrichi	Gilled	1
Hypochnicium albostramineum	Resupinate	4
Hypochnicium bombycinum	Resupinate	3
Hypochnicium polonese	Resupinate	1
Hypochnicium punctulatum	Resupinate	11
Hypochnicium subrigescens	Resupinate	3
Hypochnicium wakefieldiae	Resupinate	6
Hypomyces rosellus	Resupinate	1
Hypomyces semitranslucens	Resupinate	4
Hypoxylon fuscum	Stromatoid	1
Hypoxylon rubiginosum	Stromatoid	9
Hysterium pulicare	Pyrenoid	35
Hysterographium fraxini	Pyrenoid	8
Immersiella caudata	Pyrenoid	14
Inonotus obliquus	Resupinate	5
Irpex litschaueri	Resupinate	1
Ischnoderma benzoinum	Pileate	4
Jaapia ochroleuca	Resupinate	6
Junghuhnia collabens	Resupinate	1
Junghuhnia luteoalba	Resupinate	20
Kirschsteiniothelia cf atra	Pyrenoid	2
Kuehneromyces lignicola	Gilled	3
Kuehneromyces mutabilis	Gilled	1
Kurtia argillacea	Resupinate	31
Lachnella sp1.	Discoid	1
Lachnum corticale	Discoid	30
Lachnum pudibundum	Discoid	1
Lachnum sp1.	Discoid	27
Lachnum sp2.	Discoid	3
Lachnum virgineum	Discoid	29
Laetinaeria aff uvidula	Discoid	1
Lasiosphaeria hirsuta/tuberculosa complex	Pyrenoid	30
Lasiosphaeria ovina	Pyrenoid	13
Lasiosphaeria pyramidata	Pyrenoid	1

Laxitextum bicolor	Pileate	6
Lentaria afflata	Branched	1
Lentinellus castoreus	Gilled	1
Lentinellus flabelliformis	Gilled	1
Lentinellus micheneri	Gilled	2
Lentinellus ursinus	Gilled	3
Lentinus substrictus	Pileate	1
Lentomitella cirrhosa	Pyrenoid	32
Lentomitella crinigera	Pyrenoid	22
Lentomitella tomentosa	Pyrenoid	3
Lenzites betulina	Pileate	2
Leptodontidium trabinellum	Discoid	40
Leptoporus mollis	Pileate	2
Leptosporomyces galzinii	Resupinate	3
Leptosporomyces septentrionalis	Resupinate	2
Leucogyrophana romellii	Resupinate	3
Leucogyrophana sororia	Resupinate	7
Leucoscypha leucotricha	Discoid	2
Lophiostoma cf quadrinucleatum	Pyrenoid	3
Lophiostoma compressum	Pyrenoid	3
Lophiostoma curtum	Pyrenoid	9
Lophiostoma sp1.	Pyrenoid	6
Lophiotrema boreale	Pyrenoid	12
Lophium mytilinum	Pyrenoid	45
Megacollybia platyphylla	Gilled	2
Melanomma cf fuscidulum	Pyrenoid	25
Melanomma pulvis-pyrius	Pyrenoid	33
Melanomma subdispersum	Pyrenoid	13
Melanopsamma pomiformis	Pyrenoid	4
Melanospora caprina	Pyrenoid	2
Menispora cf glauca/caesia	Pyrenoid	20
Merismodes anomala	Discoid	23
Merulius tremellosus	Pileate	3
Metulodontia nivea	Resupinate	3
Mollisia sp1.	Discoid	169
Mollisia sp2.	Discoid	17
Mollisia sp3.	Discoid	2
Mollisia sp4.	Discoid	6
Mucronella calva	Branched	25
Mustaruusu	Discoid	21
Mycena algeriensis	Gilled	1
Mycena amicta	Gilled	2
Mycena epipterygia	Gilled	23
Mycena galericulata	Gilled	5
Mycena galopus	Gilled	6
Mycena haematopus	Gilled	3
Mycena laevigata	Gilled	2
Mycena leptocephala	Gilled	2

Mycena metata/filopes	Gilled	8
Mycena rubromarginata	Gilled	23
Mycena sanguinolenta	Gilled	4
Mycena silvae-nigrae	Gilled	2
Mycena stipata	Gilled	26
Mycena tintinnabulum	Gilled	1
Mycena viridimarginata	Gilled	9
Mycoacia aurea	Resupinate	2
Mycoacia fuscoatra	Resupinate	4
Mytilinidion mytilinellum	Pyrenoid	11
Myxarium sp1.	Discoid	2
Natantiella ligneola	Pyrenoid	10
Nectria peziza	Pyrenoid	2
Nemania atropurpurea	Stromatoid	5
Nemania dark sp.	Stromatoid	5
Nemania genea	Stromatoid	1
Nemania serpens	Stromatoid	28
Neobulgaria lilacina	Discoid	19
Neodasyscypha cerina	Discoid	8
Niesslia sp.	Pyrenoid	1
Oligoporus alni	Pileate	10
Orbilia auricolor	Discoid	2
Orbilia delicatula	Discoid	122
Orbilia sp1.	Discoid	30
Orbilia sp2.	Discoid	4
Orbilia sp3.	Discoid	15
Orbilia sp4.	Discoid	24
Orbilia sp5.	Discoid	2
Orbilia sp6.	Discoid	8
Orbilia sp7.	Discoid	4
Orbilia sp8.	Discoid	1
Otidea tuomikoskii	Gilled	1
Oxyporus corticola	Resupinate	10
Panellus mitis	Gilled	1
Panellus serotinus	Gilled	2
Panus conchatus	Gilled	1
Patinellaria sanguinea	Discoid	49
Paullicorticium pearsonii	Resupinate	2
Paullicorticium seorsum	Resupinate	3
Peniophora incarnata	Resupinate	18
Peniophora laurentii	Resupinate	2
Peniophora nuda	Resupinate	3
Peniophora pithya	Resupinate	11
Peniophora polygonia	Resupinate	1
Peniophora violaceolivida	Resupinate	10
Peniophorella guttuliferum	Resupinate	4
Peniophorella pallida	Resupinate	9
Peniophorella praetermissa	Resupinate	95

Peniophorella pubera	Resupinate	17
Perenniporia subacida	Resupinate	1
Peziza cf arvernensis	Discoid	7
Pezizella sp1.	Discoid	1
Pezizella sp2.	Discoid	1
Phaeohelotium sp1.	Discoid	1
Phaeohelotium sp2.	Discoid	5
Phaeohelotium sp3.	Discoid	2
Phanerochaete calotricha	Resupinate	1
Phanerochaete laevis	Resupinate	9
Phanerochaete sordida	Resupinate	19
Phanerochaete velutina	Resupinate	20
Phellinus ferrugineofuscus	Resupinate	22
Phellinus igniarius coll	Pileate	10
Phellinus laevigatus	Resupinate	9
Phellinus lundellii	Pileate	2
Phellinus nigrolimitatus	Pileate	8
Phellinus tremulae	Pileate	15
Phellinus viticola	Pileate	30
Phialocephala piceae	Discoid	1
Phlebia centrifuga	Resupinate	2
Phlebia femsjoeensis	Resupinate	2
Phlebia lilascens coll	Resupinate	3
Phlebia livida	Resupinate	7
Phlebia radiata	Resupinate	3
Phlebia rufa	Resupinate	2
Phlebia segregata	Resupinate	9
Phlebia serialis	Resupinate	2
Phlebia subserialis	Resupinate	2
Phlebia subulata	Resupinate	7
Phlebia tuberculata	Resupinate	1
Phlebiella christiansenii	Pileate	11
Phlebiopsis gigantea	Resupinate	1
Phloeomana clavata	Gilled	2
Phloeomana hiemalis	Gilled	1
Phloeomana speirea	Gilled	1
Pholiota flammans	Gilled	1
Pholiota scamba	Gilled	4
Pholiota squarrosa	Gilled	1
Pholiota tuberculosa	Gilled	3
Piloderma bicolor	Resupinate	53
Piloderma byssinum	Resupinate	62
Piloderma olivaceum	Resupinate	8
Piloderma sp1.	Resupinate	2
Piloderma sphaerosporum	Resupinate	9
Pisorisporium sp.	Pyrenoid	14
Pitkäkyynelhuulipyr	Discoid	12
Platystomum obtectum	Pyrenoid	3

Pleurotus pulmonarius	Gilled	1
Pluteus cervinus	Gilled	18
Pluteus podospileus	Gilled	2
Pluteus semibulbosus	Gilled	2
Polydesmia pruinosa	Discoid	11
Postia caesia	Pileate	7
Postia fragilis	Pileate	5
Postia guttulata	Pileate	2
Postia leucomallella	Pileate	9
Postia ptychogaster	Resupinate	2
Postia rennyi	Resupinate	1
Postia sericeomollis	Resupinate	4
Postia tephroleuca	Pileate	10
Postia undosa	Pileate	1
Propolis farinosa	Discoid	34
Propolis sp1.	Discoid	7
Protodontia piceicola	Resupinate	1
Protodontia subgelatinosa	Resupinate	5
Protounguicularia transiens	Discoid	7
Pseudocosmospora vilior	Pyrenoid	6
Pseudographis pinicola	Discoid	2
Pseudohydnum gelatinosum	Pileate	2
Pseudoplectania nigrella	Discoid	30
Pseudotomentella flavovirens	Resupinate	1
Pseudotomentella griseopergamacea	Resupinate	5
Pseudotomentella humicola	Resupinate	1
Pseudotomentella mucidula	Resupinate	3
Pseudotomentella nigra	Resupinate	2
Pseudotomentella tristis	Resupinate	6
Psilocistella cf conincola	Discoid	1
Psilocistella obsoleta	Discoid	1
Psilocistella sp tummakarva	Discoid	1
Psilocistella sp2.	Discoid	1
Psilocistella sp3.	Discoid	3
Psilocistella sp4.	Discoid	1
Psilocistella sp5.	Discoid	3
Psilocistella sp6.	Discoid	1
Pycnoporellus fulgens	Pileate	3
Radulomyces confluens	Resupinate	1
Rectipilus fasciculatus	Discoid	1
Repetobasidium vile	Resupinate	1
Resinicium bicolor	Resupinate	48
Resinicium furfuraceum	Resupinate	44
Resupinatus poriaeformis	Resupinate	3
Rhizochaete sulphurina	Resupinate	4
Rhizochaete violascens	Resupinate	8
Rhizoctonia fusisporus	Resupinate	6
Rhizoctonia ochracea	Resupinate	1

Rhizoctonia pseudocornigerum	Resupinate	1
Rhodonia placenta	Resupinate	3
Roridomyces roridus	Gilled	1
Ruskeapaksuseinäitiö	Pyrenoid	5
Schizospora paradoxa	Resupinate	1
Scopuloides rimosa	Resupinate	6
Scutellinia scutellata	Discoid	8
Scytinostroma galactinum	Resupinate	2
Scytinostromella heterogenea	Resupinate	1
Sebacina grisea	Resupinate	1
Serpula himantioides	Resupinate	11
Sidera lunata	Resupinate	2
Simocybe centunculus	Gilled	8
Simocybe haustellaris	Gilled	3
Sistotrema aff binucleosporum	Resupinate	2
Sistotrema aff farinaceum	Resupinate	1
Sistotrema brinkmannii	Resupinate	40
Sistotrema coroniferum	Resupinate	1
Sistotrema coronilla	Resupinate	1
Sistotrema octosporum coll	Resupinate	8
Sistotrema porulosum	Resupinate	3
Sistotrema raduloides	Resupinate	6
Sistotrema resinicystidium	Resupinate	7
Sistotrema sernanderi	Resupinate	5
Sistotrema sp nov.	Resupinate	1
Sistotremastrum suecicum	Resupinate	6
Sistotremella perpusilla	Resupinate	1
Skeletocutis amorpha	Pileate	5
Skeletocutis biguttulata	Resupinate	20
Skeletocutis brevispora	Resupinate	5
Skeletocutis carneogrisea	Pileate	4
Skeletocutis kuehneri	Resupinate	6
Skeletocutis nivea	Pileate	2
Skeletocutis papyracea/subincarnata	Resupinate	18
Skeletocutis stellae	Resupinate	1
Sordariales sp1.	Pyrenoid	1
Sphaerobasidium minutum	Resupinate	2
Sphaerostilbella berkeleyana	Resupinate	1
Steccherinum lacerum	Resupinate	1
Steccherinum ochraceum	Resupinate	1
Stereum hirsutum	Pileate	14
Stereum rugosum	Pileate	6
Stereum sanguinolentum	Pileate	1
Stereum subtomentosum	Pileate	1
Stictis cf mollis	Discoid	4
Stictis sp1.	Discoid	2
Strossmayeria basitricha	Discoid	1
Strossmayeria nigra	Discoid	2

Stypella dubia	Resupinate	1
Stypella vermiformis	Resupinate	1
Subulicystidium longisporum	Resupinate	25
Suillosporium cystidiatum	Resupinate	1
Tapinella panuoides	Gilled	1
Tomentella badia	Resupinate	1
Tomentella botryoides	Resupinate	1
Tomentella brevispina	Resupinate	3
Tomentella bryophila	Resupinate	17
Tomentella cinerascens	Resupinate	5
Tomentella coerulea	Resupinate	1
Tomentella ellisii	Resupinate	3
Tomentella lapida	Resupinate	25
Tomentella lateritia	Resupinate	3
Tomentella lilacinogrisea	Resupinate	7
Tomentella sp1.	Resupinate	1
Tomentella sp2.	Resupinate	1
Tomentella stuposa	Resupinate	4
Tomentella sublilacina	Resupinate	19
Tomentella terrestris	Resupinate	7
Tomentella umbrinospora	Resupinate	1
Tomentella viridescens	Resupinate	2
Tomentella viridula	Resupinate	1
Tomentellopsis bresadolana	Resupinate	1
Tomentellopsis cf submollis	Resupinate	1
Tomentellopsis echinospora	Resupinate	1
Tomentellopsis nigra	Resupinate	3
Tomentellopsis sp1.	Resupinate	2
Trametes hirsuta	Pileate	3
Trametes ochracea	Pileate	11
Trametes pubescens	Pileate	2
Trechispora alnicola	Resupinate	1
Trechispora byssinella	Resupinate	4
Trechispora cohaerens	Resupinate	2
Trechispora farinacea	Resupinate	16
Trechispora hymenocystis	Resupinate	12
Trechispora kavinioides	Resupinate	2
Trechispora laevis	Resupinate	4
Trechispora microspora	Resupinate	7
Trechispora minima	Resupinate	2
Trechispora stellulata	Resupinate	3
Tremella foliacea	Branched	1
Tretomyces cf microsporus	Resupinate	1
Trichaptum abietinum	Pileate	37
Trichoderma minutisporum/pachybasioides	Stromatoid	3
Trichoderma pulvinatum	Stromatoid	14
Trichoderma strictipile	Stromatoid	1
Trichoderma viride	Stromatoid	4

Tricholomopsis decora	Gilled	4
Trichophaeopsis bicuspis	Discoid	1
Trichosphaeria notabilis	Pyrenoid	1
Tubaria conspersa	Gilled	6
Tubaria furfuracea	Gilled	7
Tubulicrinis accedens	Resupinate	8
Tubulicrinis angustus	Resupinate	1
Tubulicrinis borealis	Resupinate	43
Tubulicrinis calothtrix	Resupinate	34
Tubulicrinis chaetophorus	Resupinate	1
Tubulicrinis glebulosus	Resupinate	12
Tubulicrinis medius	Resupinate	15
Tubulicrinis propinquus	Resupinate	1
Tubulicrinis sororius	Resupinate	3
Tubulicrinis strangulatus	Resupinate	15
Tubulicrinis subulatus	Resupinate	59
Tulasnella albida	Resupinate	2
Tulasnella allantospora	Resupinate	1
Tulasnella brinkmannii	Resupinate	1
Tulasnella cf conidiata	Resupinate	2
Tulasnella cystidiophora	Resupinate	4
Tulasnella eichleriana	Resupinate	11
Tulasnella fuscoviolacea	Resupinate	1
Tulasnella pallida	Resupinate	1
Tulasnella subglobospora	Resupinate	1
Tulasnella tomaculum	Resupinate	1
Tulasnella violea	Resupinate	17
Tylospora asterophora	Resupinate	4
Tylospora fibrillosa	Resupinate	41
Tympanis sp1.	Discoid	6
Urceolella sp nov.	Discoid	1
Vaginatispora cf fuckelii	Pyrenoid	14
Wallrothiella congregata	Pyrenoid	1
Vararia investiens	Resupinate	1
Veluticeps abietina	Pileate	3
Xenasma pulverulentum	Resupinate	1
Xenasma rimicola	Resupinate	1
Xenasma tulasnelloideum	Resupinate	2
Xenasmatella borealis	Resupinate	1
Xenasmatella subflavidocrisea	Resupinate	1
Xenasmatella vaga	Resupinate	55
Xenolachne longicornis	Discoid	2
Xeromphalina campanella	Gilled	2
Xeromphalina picta	Gilled	1
Xylodon asperus	Resupinate	21
Xylodon borealis	Resupinate	1
Xylodon brevisetus	Resupinate	66
Xylodon detriticus	Resupinate	9

Xylodon nespori	Resupinate	1
Xylodon radula	Resupinate	3
Xylodon rimosissimus	Resupinate	3
Xylodon sambuci	Resupinate	3
