# Supplementary material for: Wood-inhabiting fungal responses to forest naturalness vary among morpho-groups
Source: Sci Rep. 2021 Jul 16;11:14585. doi: 10.1038/s41598-021-93900-7 (PMC8285386; doi:10.1038/s41598-021-93900-7)
Supplement: Supplementary file 3 — Supplementary Table S2. [file 41598_2021_93900_MOESM3_ESM.pdf]

Wood-inhabiting fungal responses to forest naturalness vary among morpho-groups

Supplementary Table S2

Purhonen Jenna, Abrego Nerea, Komonen Atte, Huhtinen Seppo, Kotiranta Heikki, Læssøe Thomas & Halme Panu

Table S2. The generalized linear model output for site level species richness. The estimates, standard errors, Z-values and P-values are given for the explanatory variables of naturalness index and averaged log volume. Asterisk indicate P-values as follows: *** = P ≤ 0.000, ** = 0.000 < P ≤ 0.01, * = 0.01 < P ≤ 0.05, · = 0.05 < P ≤ 0.1.

All fungi on all trees	Estimate	Std. Error	Z value	P value	
(Intercept)	4.148	0.043	96.060	< 0.000	***
index	0.005	0.002	2.630	0.008	**
volume	0.207	0.039	5.340	< 0.000	***
Gilled on all trees	Estimate	Std. Error	Z value	P value	
(Intercept)	1.322	0.168	7.880	< 0.000	***
index	0.004	0.007	0.536	0.592	
volume	0.391	0.144	2.706	0.007	**
Discoid on all trees	Estimate	Std. Error	Z value	P value	
(Intercept)	2.875	0.085	33.760	< 0.000	***
index	0.006	0.004	1.730	0.084	.
volume	0.048	0.079	0.600	0.546	
Pileate on all trees	Estimate	Std. Error	Z value	P value	
(Intercept)	1.255	0.169	7.442	< 0.000	***
index	0.010	0.007	1.353	0.176	
volume	0.340	0.145	2.350	0.019	*
Pyrenoid on all trees	Estimate	Std. Error	Z value	P value	
(Intercept)	2.103	0.126	16.665	< 0.000	***
index	0.012	0.005	2.173	0.030	*
volume	-0.102	0.119	-0.852	0.395	
Branched on all trees	Estimate	Std. Error	Z value	P value	
(Intercept)	-0.095	0.418	-2.263	0.024	*
index	0.001	0.017	0.085	0.933	
volume	1.053	0.312	3.369	0.000	***
Resupinate on all trees	Estimate	Std. Error	Z value	P value	
(Intercept)	3.371	0.064	52.910	< 0.000	***
index	0.002	0.003	0.720	0.474	
volume	0.273	0.057	4.810	0.000	***
Stromatoid on all trees	Estimate	Std. Error	Z value	P value	
(Intercept)	0.090	0.288	0.312	0.755	
index	0.004	0.012	0.328	0.743	
volume	0.600	0.236	2.542	0.011	*
All fungi on birch	Estimate	Std. Error	Z value	P value	
(Intercept)	4.422	0.088	50.480	< 0.000	***
index	-0.001	0.004	-0.200	0.843	
volume	0.195	0.170	1.150	0.251	
Gilled on birch	Estimate	Std. Error	Z value	P value	
(Intercept)	2.037	0.343	5.935	0.000	***
index	0.000	0.015	0.028	0.978	
volume	-0.649	0.726	-0.893	0.372	
Discoid on birch	Estimate	Std. Error	Z value	P value	
(Intercept)	3.241	0.163	19.837	< 0.000	***
index	0.002	0.007	0.356	0.722	
volume	-0.078	0.324	-0.241	0.809	
Pileate on birch	Estimate	Std. Error	Z value	P value	
(Intercept)	1.299	0.356	3.650	0.000	***
index	0.001	0.015	0.080	0.936	
volume	0.860	0.638	1.347	0.178	
Pyrenoid on birch	Estimate	Std. Error	Z value	P value	
(Intercept)	2.644	0.220	12.017	< 0.000	***

index	0.007	0.009	0.813	0.416	
volume	-0.266	0.440	-0.604	0.546	
Branched on birch	Estimate	Std. Error	Z value	P value	
(Intercept)	-3.287	1.545	-2.128	0.033	*
index	0.016	0.055	0.282	0.778	
volume	4.126	1.793	1.301	0.021	*
Resupinate on birch	Estimate	Std. Error	Z value	P value	
(Intercept)	3.455	0.140	24.598	<0.000	***
index	-0.006	0.006	-1.063	0.288	
volume	0.456	0.270	1.690	0.091	.
Stromatoid on birch	Estimate	Std. Error	Z value	P value	
(Intercept)	0.821	0.483	1.698	0.090	.
index	-0.008	0.021	-0.367	0.714	
volume	0.868	0.891	0.974	0.330	
All fungi on spruce	Estimate	Std. Error	Z value	P value	
(Intercept)	4.068	0.094	43.100	< 0.000	***
index	0.005	0.004	1.270	0.202	
volume	0.221	0.082	2.700	0.007	**
Gilled on spruce	Estimate	Std. Error	Z value	P value	
(Intercept)	0.991	0.341	2.906	0.004	**
index	0.011	0.013	0.836	0.403	
volume	0.599	0.277	2.158	0.031	*
Discoid on spruce	Estimate	Std. Error	Z value	P value	
(Intercept)	2.483	0.219	11.346	< 0.000	***
index	0.005	0.009	0.628	0.530	
volume	0.113	0.194	0.582	0.560	
Pileate on spruce	Estimate	Std. Error	Z value	P value	
(Intercept)	2.034	0.298	6.821	0.000	***
index	0.012	0.012	1.052	0.293	
volume	-0.193	0.277	-0.695	0.487	
Pyrenoid on spruce	Estimate	Std. Error	Z value	P value	
(Intercept)	1.353	0.335	4.042	0.000	***
index	0.013	0.013	0.968	0.333	
volume	0.243	0.288	0.844	0.399	
Branched on spruce	Estimate	Std. Error	Z value	P value	
(Intercept)	0.199	0.737	0.270	0.787	
index	-0.024	0.029	-0.811	0.417	
volume	0.534	0.611	0.874	0.382	
Resupinate on spruce	Estimate	Std. Error	Z value	P value	
(Intercept)	3.445	0.131	26.334	< 0.000	***
index	0.002	0.005	0.376	0.707	
volume	0.247	0.113	2.181	0.029	*
Stromatoid on spruce	Estimate	Std. Error	Z value	P value	
(Intercept)	-0.393	0.752	-0.523	0.601	
index	-0.003	0.029	-0.096	0.924	
volume	0.675	0.610	1.107	0.268	
All fungi on pine	Estimate	Std. Error	Z value	P value	
(Intercept)	3.793	0.101	37.580	< 0.000	***
index	0.009	0.004	2.160	0.031	*
volume	0.339	0.123	2.770	0.006	**
Gilled on pine	Estimate	Std. Error	Z value	P value	
(Intercept)	1.337	0.358	3.733	0.000	***
index	0.007	0.016	0.448	0.654	
volume	0.273	0.442	0.617	0.537	
Discoid on pine	Estimate	Std. Error	Z value	P value	
(Intercept)	2.401	0.227	10.575	0.000	***
index	0.015	0.009	1.599	0.110	.
volume	-0.185	0.276	-0.670	0.503	
Pileate on pine	Estimate	Std. Error	Z value	P value	
(Intercept)	0.690	0.477	1.448	0.147	

index	0.007	0.021	0.351	0.725	
volume	0.388	0.584	0.665	0.506	
Pyrenoid on pine	Estimate	Std. Error	Z value	P value	
(Intercept)	1.364	0.343	3.981	0.000	***
index	0.022	0.014	1.599	0.110	
volume	-0.010	0.395	-0.025	0.980	
Branched on pine	Estimate	Std. Error	r Z value	P value	
(Intercept)	-0.894	0.891	-1.004	0.316	
index	0.006	0.040	0.152	0.879	
volume	0.973	1.072	0.907	0.364	
Resupinate on pine	Estimate	Std. Error	Z value	P value	
(Intercept)	3.167	0.133	23.763	< 0.000	***
index	0.004	0.006	0.800	0.424	
volume	0.572	0.164	3.488	0.001	***
Stromatoid on pine	Estimate	Std. Error	Z value	P value	
(Intercept)	-3.335	1.940	-1.719	0.086	.
index	0.085	0.064	1.335	0.182	
volume	0.393	1.448	0.271	0.786	
All fungi on aspen	Estimate	Std. Error	Z value	P value	
(Intercept)	4.156	0.083	50.030	< 0.000	***
index	0.005	0.004	1.310	0.191	
volume	0.266	0.061	4.390	< 0.000	***
Gilled on aspen	Estimate	Std. Error	Z value	P value	
(Intercept)	0.833	0.387	2.152	0.031	*
index	-0.003	0.016	-0.208	0.835	
volume	0.808	0.259	3.126	0.002	**
Discoid on aspen	Estimate	Std. Error	Z value	P value	
(Intercept)	3.150	0.143	22.088	<0.000	***
index	0.007	0.006	1.155	0.248	
volume	0.095	0.107	0.892	0.372	
Pileate on aspen	Estimate	Std. Error	Z value	P value	
(Intercept)	0.623	0.390	1.598	0.110	
index	0.017	0.015	1.121	0.262	
volume	0.636	0.257	2.474	0.013	*
Pyrenoid on aspen	Estimate	Std. Error	Z value	P value	
(Intercept)	2.236	0.226	9.906	<0.000	***
index	0.005	0.010	0.474	0.636	
volume	0.147	0.169	0.870	0.384	
Branched on aspen	Estimate	Std. Error	Z value	P value	
(Intercept)	-1.553	0.945	-1.645	0.100	
index	0.022	0.033	0.662	0.508	
volume	1.102	0.575	1.916	0.055	.
Resupinate on aspen	Estimate	Std. Error	Z value	P value	
(Intercept)	3.282	0.132	24.809	<0.000	***
index	0.002	0.006	0.293	0.770	
volume	0.248	0.098	2.546	0.011	*
Stromatoid on aspen	Estimate	Std. Error	Z value	P value	
(Intercept)	0.246	0.493	0.499	0.618	
index	0.005	0.020	0.232	0.817	
volume	0.784	0.326	2.408	0.016	*
