# Supplementary material for: Wood-inhabiting fungal responses to forest naturalness vary among morpho-groups
Source: Sci Rep. 2021 Jul 16;11:14585. doi: 10.1038/s41598-021-93900-7 (PMC8285386; doi:10.1038/s41598-021-93900-7)
Supplement: Supplementary file 4 — Supplementary Table S3. [file 41598_2021_93900_MOESM4_ESM.pdf]

Wood-inhabiting fungal responses to forest naturalness vary among morpho-groups

Supplementary Table S3

Purhonen Jenna, Abrego Nerea, Komonen Atte, Huhtinen Seppo, Kotiranta Heikki, Læssøe Thomas & Halme Panu

Table S3. The generalized linear mixed model output of log level species richness. The estimates, standard errors, Z-values and P-values are given for the standardized explanatory variables of log bark cover (SD = 33.00), decay stage (0.54), naturalness index (9.00), moss cover (12.88), log volume (1.25) and log volume squared (1.66). Asterisk indicate P-values as follows: *** = $P \le 0.000$, ** = $0.000 < P \le 0.01$, * = $0.01 < P \le 0.05$, · = $0.05 < P \le 0.1$.

All fungi on all trees	Estimate	Std. Error	Z value	P value	
(Intercept)	3.390	0.019	176.650	< 0.000	***
Bark	0.033	0.014	2.330	0.020	*
Decay	-0.024	0.015	-1.650	0.099	.
Index	0.049	0.019	2.520	0.012	*
Moss	0.016	0.015	1.100	0.273	
Volume	0.224	0.038	5.870	< 0.000	***
Volume2	-0.124	0.036	-3.470	0.001	***
**Gilled on all trees**	**Estimate**	**Std. Error**	**Z value**	**P value**	
(Intercept)	0.466	0.069	6.713	< 0.000	***
Bark	0.038	0.060	0.631	0.528	
Decay	-0.010	0.061	-0.167	0.867	
Index	0.038	0.068	0.551	0.582	
Moss	0.223	0.061	3.649	0.000	***
Volume	0.397	0.148	2.682	0.007	**
Volume2	-0.182	0.132	-1.382	0.167	
**Discoid on all trees**	**Estimate**	**Std. Error**	**Z value**	**P value**	
(Intercept)	2.150	0.025	86.880	< 0.000	***
Bark	0.044	0.025	1.750	0.080	.
Decay	-0.094	0.027	-3.440	0.001	***
Index	0.044	0.025	1.770	0.077	.
Moss	-0.029	0.028	-1.050	0.295	
Volume	0.187	0.068	2.760	0.006	**
Volume2	-0.126	0.065	-1.930	0.054	.
**Pileate on all trees**	**Estimate**	**Std. Error**	**Z value**	**P value**	
(Intercept)	0.686	0.074	9.284	< 0.000	***
Bark	0.340	0.048	7.115	< 0.000	***
Decay	-0.074	0.052	-1.433	0.152	
Index	0.085	0.072	1.173	0.241	
Moss	0.037	0.055	0.679	0.497	
Volume	0.444	0.130	3.416	0.001	***
Volume2	-0.206	0.108	-1.900	0.057	.
**Pyrenoid on all trees**	**Estimate**	**Std. Error**	**Z value**	**P value**	
(Intercept)	1.165	0.041	28.526	< 0.000	***
Bark	0.174	0.039	4.488	< 0.000	***
Decay	-0.005	0.041	-0.121	0.904	
Index	0.092	0.040	2.282	0.023	*
Moss	-0.136	0.045	-2.999	0.003	**
Volume	-0.007	0.104	-0.063	0.950	
Volume2	0.043	0.092	0.467	0.641	
**Branched on all trees**	**Estimate**	**Std. Error**	**Z value**	**P value**	

(Intercept)	-1.362	0.151	-8.998	< 0.000	***
Bark	0.023	0.146	0.157	0.876	
Decay	0.062	0.142	0.436	0.663	
Index	0.070	0.135	0.519	0.604	
Moss	-0.128	0.156	-0.820	0.412	
Volume	1.007	0.321	3.135	0.002	**
Volume2	-0.509	0.277	-1.840	0.066	.
Resupinate on all trees	Estimate	Std. Error	Z value	P value	
(Intercept)	2.561	0.023	112.790	< 0.000	***
Bark	-0.080	0.022	-3.640	0.000	***
Decay	0.039	0.022	1.770	0.077	.
Index	0.034	0.023	1.480	0.138	
Moss	0.054	0.022	2.440	0.014	*
Volume	0.291	0.061	4.780	0.000	***
Volume2	-0.221	0.061	-3.630	0.000	***
Stromatoid on all trees	Estimate	Std. Error	Z value	P value	
(Intercept)	-0.603	0.121	-4.998	< 0.000	***
Bark	0.084	0.098	0.857	0.391	
Decay	-0.367	0.116	-3.166	0.002	**
Index	0.059	0.115	0.513	0.608	
Moss	0.038	0.108	0.349	0.727	
Volume	0.227	0.240	0.947	0.343	
Volume2	-0.036	0.201	-0.178	0.859	
All fungi on birch	Estimate	Std. Error	Z value	P value	
(Intercept)	3.582	0.078	46.000	< 0.000	***
Bark	-0.014	0.030	-0.460	0.645	
Decay	-0.079	0.028	-2.800	0.005	**
Index	-0.023	0.039	-0.590	0.556	
Moss	-0.020	0.040	-0.510	0.607	
Volume	0.100	0.222	0.450	0.651	
Volume2	0.199	0.420	0.470	0.637	
Gilled on birch	Estimate	Std. Error	Z value	P value	
(Intercept)	0.179	0.345	0.518	0.604	
Bark	0.111	0.123	0.900	0.368	
Decay	-0.148	0.120	-1.230	0.219	
Index	-0.106	0.123	-0.866	0.386	
Moss	-0.071	0.166	-0.427	0.670	
Volume	1.248	0.983	1.269	0.204	
Volume2	-2.081	1.885	-1.104	0.270	
Discoid on birch	Estimate	Std. Error	Z value	P value	
(Intercept)	2.539	0.114	22.197	< 0.000	***
Bark	-0.035	0.047	-0.743	0.457	
Decay	-0.054	0.046	-1.182	0.237	
Index	-0.014	0.049	-0.297	0.767	
Moss	0.011	0.064	0.168	0.867	
Volume	0.035	0.367	0.097	0.923	
Volume2	0.343	0.684	0.501	0.617	
Pileate on birch	Estimate	Std. Error	Z value	P value	
(Intercept)	1.044	0.229	4.564	< 0.000	***
Bark	0.069	0.100	0.693	0.489	

Decay	-0.231	0.106	-2.173	0.030	*
Index	-0.028	0.103	-0.272	0.786	
Moss	-0.115	0.151	-0.763	0.445	
Volume	-0.670	0.744	-0.902	0.367	
Volume2	1.943	1.326	1.465	0.143	

Pyrenoid on birch	Estimate	Std. Error	Z value	P value	
(Intercept)	1.493	0.188	7.935	< 0.000	***
Bark	0.048	0.072	0.670	0.503	
Decay	0.033	0.061	0.546	0.585	
Index	0.060	0.069	0.871	0.384	
Moss	-0.063	0.090	-0.701	0.483	
Volume	0.047	0.539	0.088	0.930	
Volume2	-0.197	1.052	-0.187	0.851	

Branched on birch	Estimate	Std. Error	Z value	P value	
(Intercept)	-1.203	0.813	-1.480	0.139	
Bark	-0.493	0.451	-1.095	0.274	
Decay	-0.411	0.638	-0.644	0.520	
Index	0.117	0.439	-0.267	0.789	
Moss	0.054	0.741	0.073	0.942	
Volume	-1.008	2.759	-0.365	0.715	
Volume2	3.227	4.515	0.715	0.475	

Resupinate on birch	Estimate	Std. Error	Z value	P value	
(Intercept)	2.613	0.107	24.436	< 0.000	***
Bark	-0.033	0.045	-0.719	0.472	
Decay	-0.072	0.045	-1.603	0.109	
Index	0.053	0.046	1.134	0.257	
Moss	0.011	0.061	0.172	0.864	
Volume	0.132	0.340	0.389	0.697	
Volume2	0.231	0.627	0.368	0.713	

Stromatoid on birch	Estimate	Std. Error	Z value	P value	
(Intercept)	-0.011	0.387	-0.03	0.976	
Bark	-0.199	0.165	-1.204	0.229	
Decay	-0.271	0.203	-1.333	0.183	
Index	0.038	0.171	0.22	0.826	
Moss	-0.118	0.267	-0.443	0.657	
Volume	-0.024	1.218	-0.02	0.984	
Volume2	0.471	2.178	0.216	0.829	

All fungi on spruce	Estimate	Std. Error	Z value	P value	
(Intercept)	3.335	0.034	99.320	< 0.000	***
Bark	-0.040	0.032	-1.260	0.207	
Decay	0.071	0.030	2.330	0.020	*
Index	0.086	0.028	3.140	0.002	**
Moss	0.019	0.032	0.590	0.556	
Volume	0.123	0.098	1.260	0.208	
Volume2	-0.030	0.089	-0.330	0.739	

Gilled on spruce	Estimate	Std. Error	Z value	P value	
(Intercept)	0.361	0.152	2.381	0.017	*
Bark	0.018	0.129	0.136	0.892	
Decay	-0.022	0.125	-0.180	0.857	
Index	0.156	0.110	1.421	0.155	

Moss	0.319	0.132	2.420	0.016	*
Volume	0.300	0.379	0.792	0.428	
Volume2	-0.017	0.327	-0.051	0.959	
Discoid on spruce	Estimate	Std. Error	Z value	P value	
(Intercept)	1.842	0.070	26.315	< 0.000	***
Bark	-0.046	0.070	-0.653	0.514	
Decay	0.078	0.066	1.187	0.235	
Index	0.082	0.059	1.378	0.168	
Moss	-0.146	0.070	-2.085	0.037	*
Volume	0.077	0.214	0.359	0.720	
Volume2	-0.008	0.196	-0.042	0.967	
Pileate on spruce	Estimate	Std. Error	Z value	P value	
(Intercept)	1.186	0.099	12.017	< 0.000	***
Bark	0.152	0.084	1.812	0.070	·
Decay	-0.037	0.082	-0.456	0.648	
Index	0.143	0.078	1.828	0.068	·
Moss	0.124	0.086	1.449	0.147	
Volume	0.463	0.278	1.669	0.095	·
Volume2	-0.314	0.258	-1.218	0.223	
Pyrenoid on spruce	Estimate	Std. Error	Z value	P value	
(Intercept)	0.675	0.127	5.311	< 0.000	***
Bark	0.085	0.117	0.724	0.469	
Decay	0.149	0.113	1.320	0.187	
Index	0.153	0.108	1.412	0.158	
Moss	0.003	0.121	0.023	0.981	
Volume	-0.434	0.377	-1.149	0.250	
Volume2	0.452	0.337	1.340	0.180	
Branched on spruce	Estimate	Std. Error	Z value	P value	
(Intercept)	-1.297	0.355	-3.652	0.000	***
Bark	0.614	0.241	2.545	0.011	*
Decay	0.182	0.235	0.776	0.438	
Index	-0.115	0.217	-0.533	0.594	
Moss	-0.285	0.246	-1.158	0.247	
Volume	0.977	0.813	1.201	0.230	
Volume2	-0.343	0.681	-0.503	0.615	
Resupinate on spruce	Estimate	Std. Error	Z value	P value	
(Intercept)	2.648	0.049	54.240	< 0.000	***
Bark	-0.155	0.047	-3.280	0.001	***
Decay	0.106	0.044	2.410	0.016	*
Index	0.071	0.041	1.760	0.079	.
Moss	0.026	0.046	0.570	0.566	
Volume	0.095	0.143	0.670	0.505	
Volume2	-0.049	0.128	-0.380	0.701	
Stromatoid on spruce	Estimate	Std. Error	Z value	P value	
(Intercept)	-1.367	0.353	-3.873	0.000	***
Bark	0.286	0.303	0.944	0.345	
Decay	-0.243	0.302	-0.804	0.422	
Index	-0.006	0.271	-0.022	0.982	
Moss	0.280	0.314	0.891	0.373	
Volume	-0.235	0.973	-0.241	0.810	

Volume2	0.543	0.834	0.651	0.515	
All fungi on pine	Estimate	Std. Error	Z value	P value	
(Intercept)	3.337	0.155	21.464	< 0.000	***
Bark	0.195	0.216	0.901	0.367	
Decay	0.013	0.038	0.356	0.722	
Index	0.049	0.032	1.527	0.127	
Moss	0.082	0.034	2.420	0.016	*
Volume	0.567	0.171	3.317	0.001	***
Volume2	-0.538	0.246	-2.186	0.029	*
Gilled on pine	Estimate	Std. Error	Z value	P value	
(Intercept)	0.159	0.573	0.276	0.782	
Bark	-0.284	0.783	-0.362	0.717	
Decay	0.240	0.150	1.599	0.110	
Index	-0.138	0.138	-0.999	0.318	
Moss	0.474	0.133	3.568	0.000	***
Volume	0.555	0.676	0.820	0.412	
Volume2	-0.356	0.945	-0.377	0.706	
Discoid on pine	Estimate	Std. Error	Z value	P value	
(Intercept)	1.667	0.336	4.958	< 0.000	***
Bark	-0.067	0.464	-0.145	0.885	
Decay	-0.083	0.079	-1.047	0.295	
Index	0.069	0.066	1.047	0.295	
Moss	0.066	0.071	0.928	0.353	
Volume	0.439	0.365	1.203	0.229	
Volume2	-0.459	0.533	-0.863	0.388	
Pileate on pine	Estimate	Std. Error	Z value	P value	
(Intercept)	0.615	0.807	0.762	0.446	
Bark	1.117	1.142	0.978	0.328	
Decay	-0.095	0.207	-0.458	0.647	
Index	0.121	0.171	0.708	0.479	
Moss	-0.137	0.186	-0.737	0.461	
Volume	1.100	0.876	1.256	0.209	
Volume2	-0.974	1.250	-0.779	0.436	
Pyrenoid on pine	Estimate	Std. Error	Z value	P value	
(Intercept)	1.654	0.507	3.262	0.001	**
Bark	1.271	0.727	1.749	0.080	.
Decay	-0.032	0.128	-0.247	0.805	
Index	0.192	0.106	1.815	0.070	.
Moss	-0.075	0.119	-0.630	0.529	
Volume	0.021	0.568	0.036	0.971	
Volume2	0.096	0.822	0.116	0.907	
Branched on pine	Estimate	Std. Error	Z value	P value	
(Intercept)	0.216	1.472	0.147	0.883	
Bark	2.302	2.129	1.081	0.279	
Decay	0.016	0.383	0.041	0.967	
Index	-0.049	0.316	-0.154	0.877	
Moss	-0.540	0.358	-1.508	0.131	
Volume	0.892	1.548	0.576	0.564	
Volume2	-0.489	2.223	-0.220	0.826	
Resupinate on pine	Estimate	Std. Error	Z value	P value	

(Intercept)	2.700	0.208	13.011	< 0.000	***
Bark	0.109	0.287	0.380	0.704	
Decay	0.047	0.050	0.947	0.344	
Index	0.027	0.043	0.620	0.535	
Moss	0.090	0.045	2.002	0.045	*
Volume	0.684	0.227	3.006	0.003	**
Volume2	-0.684	0.327	-2.095	0.036	*
Stromatoid on pine	Estimate	Std. Error	Z value	P value	
(Intercept)	-8.296	5.628	-1.474	0.140	
Bark	-7.227	7.074	-1.022	0.307	
Decay	-0.797	0.688	-1.158	0.247	
Index	0.744	0.593	1.254	0.210	
Moss	0.021	0.703	0.029	0.977	
Volume	-1.686	3.025	-0.557	0.577	
Volume2	2.083	4.522	0.461	0.645	
All fungi on aspen	Estimate	Std. Error	Z value	P value	
(Intercept)	3.383	0.046	74.160	< 0.000	***
Bark	-0.035	0.049	-0.710	0.475	
Decay	-0.055	0.038	-1.460	0.145	
Index	0.068	0.038	1.780	0.075	.
Moss	0.069	0.034	2.020	0.044	*
Volume	0.168	0.067	2.500	0.013	*
Volume2	-0.099	0.057	-1.740	0.081	.
Gilled on aspen	Estimate	Std. Error z	Z value	P value	
(Intercept)	-0.062	0.197	-0.314	0.754	
Bark	-0.242	0.193	-1.252	0.211	
Decay	-0.351	0.175	-2.008	0.045	*
Index	0.010	0.130	0.078	0.938	
Moss	0.302	0.147	2.059	0.040	*
Volume	0.487	0.247	1.975	0.048	*
Volume2	-0.177	0.216	-0.819	0.413	
Discoid on aspen	Estimate	Std. Error	Z value	P value	
(Intercept)	2.388	0.058	41.310	< 0.000	***
Bark	-0.125	0.065	-1.920	0.055	.
Decay	-0.057	0.057	-1.000	0.319	
Index	0.050	0.044	1.150	0.250	
Moss	0.066	0.053	1.230	0.217	
Volume	0.173	0.099	1.750	0.080	.
Volume2	-0.142	0.095	-1.490	0.135	
Pileate on aspen	Estimate	Std. Error	Z value	P value	
(Intercept)	0.246	0.194	1.272	0.203	
Bark	0.396	0.161	2.452	0.014	*
Decay	-0.124	0.190	-0.653	0.514	
Index	0.069	0.164	0.421	0.674	
Moss	-0.042	0.165	-0.257	0.797	
Volume	0.334	0.277	1.205	0.228	
Volume2	-0.081	0.218	-0.371	0.711	
Pyrenoid on aspen	Estimate	Std. Error	Z value	P value	
(Intercept)	1.396	0.093	15.084	< 0.000	***
Bark	0.043	0.103	0.418	0.676	

Decay	0.050	0.100	0.498	0.618	
Index	0.096	0.077	1.255	0.209	
Moss	-0.156	0.097	-1.607	0.108	
Volume	0.213	0.154	1.382	0.167	
Volume2	-0.131	0.138	-0.950	0.342	
Branched on aspen	Estimate	Std. Error	Z value	P value	
(Intercept)	-1.829	0.501	-3.649	0.000	***
Bark	-0.468	0.555	-0.842	0.400	
Decay	0.378	0.409	0.925	0.355	
Index	0.600	0.337	1.780	0.075	.
Moss	-0.039	0.395	-0.098	0.922	
Volume	0.957	0.611	1.566	0.117	
Volume2	-0.381	0.510	-0.748	0.454	
Resupinate on aspen	Estimate	Std. Error	Z value	P value	
(Intercept)	2.355	0.059	40.240	< 0.000	***
Bark	-0.085	0.065	-1.320	0.188	
Decay	-0.052	0.058	-0.900	0.367	
Index	0.065	0.045	1.460	0.144	
Moss	0.110	0.054	2.040	0.041	*
Volume	0.215	0.099	2.170	0.030	*
Volume2	-0.167	0.094	-1.780	0.075	.
Stromatoid on aspen	Estimate	Std. Error	Z value	P value	
(Intercept)	-0.073	0.210	-0.347	0.728	
Bark	-0.091	0.212	-0.426	0.670	
Decay	-0.065	0.201	-0.325	0.745	
Index	0.068	0.151	0.453	0.650	
Moss	0.176	0.178	0.986	0.324	
Volume	0.319	0.300	1.064	0.287	
Volume2	-0.147	0.263	-0.561	0.575	
