# Supplementary material for: Wood-inhabiting fungal responses to forest naturalness vary among morpho-groups
Source: Sci Rep. 2021 Jul 16;11:14585. doi: 10.1038/s41598-021-93900-7 (PMC8285386; doi:10.1038/s41598-021-93900-7)
Supplement: Supplementary file 5 — Supplementary Table S4. [file 41598_2021_93900_MOESM5_ESM.pdf]

Wood-inhabiting fungal responses to forest naturalness vary among morpho-groups

Supplementary Table S4

Purhonen Jenna, Abrego Nerea, Komonen Atte, Huhtinen Seppo, Kotiranta Heikki, Læssøe Thomas & Halme Panu

Table S4. Spearman rank correlation coefficients from Bioenv-analysis for community dissimilarities and environmental variables or their combinations. The results are represented separately for all fungi and different morpho-groups on different tree species. The highest correlation is bolded.

All fungi						Correlation
Bark						0.1354
Index	Bark					0.1557
**Index**	**Volume**	**Bark**				**0.1653**
Index	Volume	Moss	Bark			0.1600
Index	Volume	Moss	Decay	Bark		0.1538
Index	Volume	Moss	Decay	Bark	Site	0.1423
All discoid fungi						Correlation
Bark						0.1004
Volume	Bark					0.1157
Volume	Moss	Bark				0.1260
Index	Volume	Moss	Bark			0.1282
**Index**	**Volume**	**Moss**	**Decay**	**Bark**		**0.1304**
Index	Volume	Moss	Decay	Bark	Site	0.1200
All pileate fungi						Correlation
**Volume**						**0.1075**
Volume	Decay					0.1028
Volume	Decay	Bark				0.0829
Volume	Moss	Decay	Bark			0.0762
Volume	Moss	Decay	Bark	Site		0.0685
Index	Volume	Moss	Decay	Bark	Site	0.0543
All pyrenoid fungi						Correlation
Bark						0.0664
Decay	Bark					0.0798
**Moss**	**Decay**	**Bark**				**0.0857**
Index	Moss	Decay	Bark			0.0872
Index	Moss	Decay	Bark	Site		0.0845
Index	Volume	Moss	Decay	Bark	Site	0.0713
All resupinate fungi						Correlation
Bark						0.1240
Index	Bark					0.1607
**Index**	**Volume**	**Bark**				**0.1613**
Index	Volume	Decay	Bark			0.1431
Index	Volume	Moss	Decay	Bark		0.1265
Index	Volume	Moss	Decay	Bark	Site	0.1120
All fungi on birch						Correlation
Decay						0.2059
Decay	Bark					0.2313

Moss	Decay	Bark				0.2306
**Index**	**Moss**	**Decay**	**Bark**			**0.2401**
Index	Moss	Decay	Bark	Site		0.2217
Index	Volume	Moss	Decay	Bark	Site	0.11331
Discoid on birch						Correlation
Decay						0.1093
**Decay**	**Bark**					**0.1470**
Index	Decay	Bark				0.1410
Index	Moss	Decay	Bark			0.1224
Index	Moss	Decay	Bark	Site		0.1062
Index	Volume	Moss	Decay	Bark	Site	0.0745
Pileate on birch						Correlation
Decay						0.2210
**Decay**	**Bark**					**0.2932**
Moss	Decay	Bark				0.2860
Index	Moss	Decay	Bark			0.2717
Index	Moss	Decay	Bark	Site		0.2589
Index	Volume	Moss	Decay	Bark	Site	0.2179
Pyrenoid on birch						Correlation
Moss						0.2002
**Moss**	**Decay**					**0.2422**
Index	Moss	Decay				0.2086
Moss	Decay	Bark	Site			0.1923
Index	Moss	Decay	Bark	Site		0.1837
Index	Volume	Moss	Decay	Bark	Site	0.1457
Resupinate on birch						Correlation
Bark						0.1478
Decay	Bark					0.1574
**Index**	**Decay**	**Bark**				**0.1785**
Index	Moss	Decay	Bark			0.1605
Index	Moss	Decay	Bark	Site		0.1441
Index	Volume	Moss	Decay	Bark	Site	0.0727
All fungi on spruce						Correlation
Bark						0.2145
Moss	Bark					0.2998
**Moss**	**Decay**	**Bark**				**0.3194**
Index	Moss	Decay	Bark			0.2957
Index	Moss	Decay	Bark	Site		0.2776
Index	Volume	Moss	Decay	Bark	Site	0.2248
Discoid on spruce						Correlation
Moss						0.1212
Moss	Decay					0.1430
Index	Moss	Bark				0.1708
**Index**	**Moss**	**Decay**	**Bark**			**0.1838**
Index	Moss	Decay	Bark	Site		0.1824
Index	Volume	Moss	Decay	Bark	Site	0.1494
Pileate on spruce						Correlation

Bark						0.2163
**Moss**	**Bark**					**0.2228**
Moss	Decay	Bark				0.2196
Moss	Decay	Bark	Site			0.1846
Index	Moss	Decay	Bark	Site		0.1385
Index	Volume	Moss	Decay	Bark	Site	0.0754
Pyrenoid on spruce						Correlation
Volume						0.1247
Index	Volume					0.1259
**Index**	**Volume**	**Site**				**0.1382**
Index	Volume	Moss	Site			0.1382
Index	Volume	Moss	Decay	Site		0.1151
Index	Volume	Moss	Decay	Bark	Site	0.0848
Resupinate on spruce						Correlation
Bark						0.1786
Moss	Bark					0.2323
**Moss**	**Decay**	**Bark**				**0.2616**
Index	Moss	Decay	Bark			0.2258
Index	Moss	Decay	Bark	Site		0.1820
Index	Volume	Moss	Decay	Bark	Site	0.1360
All fungi on pine						Correlation
Index						0.1376
Index	Moss					0.1818
**Index**	**Moss**	**Decay**				**0.2022**
Index	Moss	Decay	Site			0.1720
Index	Moss	Decay	Bark	Site		0.1486
Index	Volume	Moss	Decay	Bark	Site	0.1370
Discoid on pine						Correlation
Index						0.1477
Index	Moss					0.2022
**Index**	**Moss**	**Decay**				**0.2279**
Index	Moss	Decay	Bark			0.1884
Index	Volume	Moss	Decay	Bark		0.1578
Index	Volume	Moss	Decay	Bark	Site	0.1342
Pyrenoid on pine						Correlation
**Site**						**0.2058**
Decay	Site					0.1838
Volume	Decay	Site				0.1721
Index	Volume	Decay	Site			0.1513
Index	Volum	Moss	Decay	Site		0.1283
Index	Volum	Moss	Decay	Bark	Site	0.0959
Resupinate on pine						Correlation
Index						0.1239
**Index**	**Moss**					**0.1287**
Index	Moss	Decay				0.1222
Index	Volume	Moss	Bark			0.1210
Index	Volume	Moss	Decay	Bark		0.1194

Index	Volume	Moss	Decay	Bark	Site	0.0891
All fungi on aspen						Correlation
Bark						0.1326
Index	Bark					0.2160
**Index**	**Volume**	**Bark**				**0.2603**
Index	Volume	Bark	Site			0.2505
Index	Volume	Moss	Bark	Site		0.2327
Index	Volume	Moss	Decay	Bark	Site	0.1923
Discoid on aspen						Correlation
Bark						0.0978
Index	Bark					0.1678
**Index**	**Volume**	**Bark**				**0.1842**
Index	Volume	Bark	Site			0.1834
Index	Volume	Moss	Bark	Site		0.1714
Index	Volume	Moss	Decay	Bark	Site	0.1302
Pyrenoid on aspen						Correlation
**Index**						**0.0724**
Index	Decay					0.0706
Index	Moss	Decay				0.0674
Index	Moss	Decay	Site			0.0585
Index	Volume	Moss	Decay	Site		0.0381
Index	Volume	Moss	Decay	Bark	Site	0.0272
Resupinate on aspen						Correlation
Bark						0.1229
Volume	Bark					0.1697
**Index**	**Volume**	**Bark**				**0.2012**
Index	Volume	Bark	Site			0.1947
Index	Volume	Moss	Bark	Site		0.1775
Index	Volume	Moss	Decay	Bark	Site	0.1464
