# Supplementary material for: Wood-inhabiting fungal responses to forest naturalness vary among morpho-groups
Source: Sci Rep. 2021 Jul 16;11:14585. doi: 10.1038/s41598-021-93900-7 (PMC8285386; doi:10.1038/s41598-021-93900-7)
Supplement: Supplementary file 6 — Supplementary Information. [file 41598_2021_93900_MOESM6_ESM.pdf]

Wood-inhabiting fungal responses to forest naturalness vary among morpho-groups

Supplementary Results

Purhonen Jenna, Abrego Nerea, Komonen Atte, Huhtinen Seppo, Kotiranta Heikki, Læssøe Thomas & Halme Panu

Exploring the environmental variables

We used Wilcoxon rank sum test to check whether the six least natural sites differ from the six most natural sites according to the measured forest level variables; total volume of dead wood per hectare, the age of the canopy trees and the number of stumps per hectare. Then, using the same test, we explored whether sites were equal considering the log level variables; volume, decay stage, bark cover and moss cover.

Log and forest level variables

The total dead wood volume tended to differ (W = 6, P-value = 0.065), and the age of the canopy trees and number of stumps differed significantly (W = 1, P-value = 0.004 and W = 35, P-value = 0.004, respectively) between the least and most natural sites (Fig. S2). The mean values of the environmental variables are given in the Table S5.

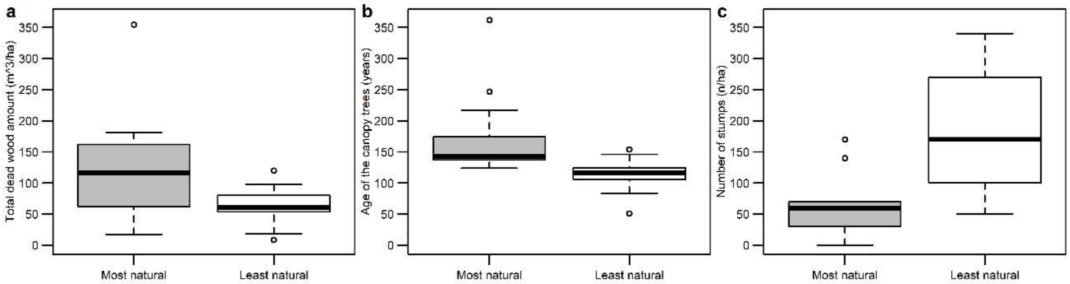

Figure S2 a) The total dead wood volume, b) the age of the canopy trees, and c) the number of stumps in the six most natural (grey) and six least natural (white). Each box presents 50% of the values, the upper and lower whiskers and dots (outliers) the upper and lower 25% of the values, respectively. The black line inside the box represents the median.

Table S5. The mean and standard error for forest level environmental variables in the six least and most managed forests

Forest naturalness	Volume(m^3/ha)±SD	Age(years)±SD	Stump(n/ha)±SD
Least natural	61.4±26.9	113.7±17.8	186.6±95.4
Most natural	136.8±105.0	167.9±55.7	68.6±55.1

The log characteristics did not differ between the least and most natural sites, except for the moss cover of birch (W = 397.5, P-value = 0.023) and volume of pine study logs (W = 197, P-value = 0.062) (Fig. S3). We want to highlight that, majority of the study logs were of decay stage 2 (N = 113) and decay stage 3 (N = 75), while only four study logs were of decay stage 4. Thus, we conclude that the quality of the study logs was relatively similar between the different study sites.

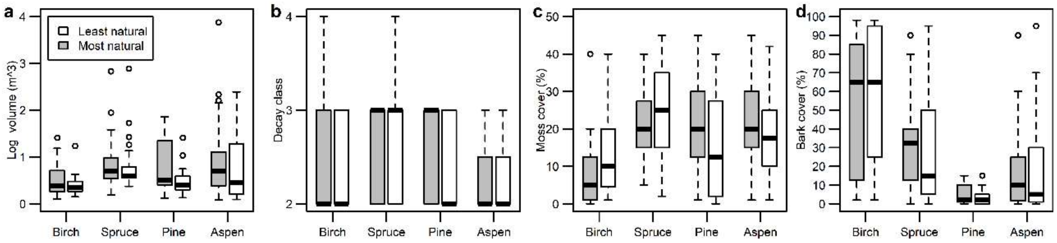

Figure S3 a) The volume, b) decay stage, c) moss cover, and d) bark cover of the studied logs for each target tree species in the most (grey) and the least (white) natural sites. Each box presents 50% of the values, the upper and lower whiskers and dots (outliers) the upper and lower 25% of the values, respectively. The black line inside the box represents the median.
