# Supplementary material for: Wood-inhabiting fungal responses to forest naturalness vary among morpho-groups
Source: Sci Rep. 2021 Jul 16;11:14585. doi: 10.1038/s41598-021-93900-7 (PMC8285386; doi:10.1038/s41598-021-93900-7)
Supplement: Supplementary file 7 — Supplementary Legends. [file 41598_2021_93900_MOESM7_ESM.docx]

**Supplementary Information captions**

**Supplementary Table S1** List of detected species or taxonomic groups in alphabetical order, their morpho-group and total abundance

**Supplementary Table S2** Table of the generalized linear model outputs for site level species richness

**Supplementary Table S3** Table of the generalized linear mixed model outputs for log level species richness

**Supplementary Table S4** Spearman rank correlation coefficients from Bioenv-analysis for community dissimilarities and environmental variables or their combinations

**Supplementary Figure S1** NMDS and Bioenv-analysis results for morpho-groups inhabiting different tree species

**Supplementary Results** Comparison of the site level and log level variables between the least and most managed sites. Includes Supplementary Figure S2 and S3, as well as Table S5
